# Microenvironment-Engineered Biocatalytic Metal–Organic Framework Nanomotors for Selective and Transformative Water Decontamination

**DOI:** 10.1007/s40820-025-02064-w

**Published:** 2026-01-26

**Authors:** Shu Xu, Jueyi Xue, Linyun Bao, Joel Yong, Ying Cao, Jun Ma, Kang Liang

**Affiliations:** 1 SCNU Environmental Research Institute, Guangdong Provincial Key Laboratory of Chemical Pollution and Environmental Safety and MOE Key Laboratory of Theoretical Chemistry of Environment, School of Environment, South China Normal University, University Town, Guangzhou, 510006 People’s Republic of China; 2 School of Chemical Engineering and Graduate School of Biomedical Engineering, The University of New South Wales, Sydney, NSW 2052 Australia; 3 State Key Laboratory of Urban Water Resource and Environment, School of Environment, Harbin Institute of Technology, Harbin, 150090 People’s Republic of China; 4 State Key Laboratory of Advanced Environmental Technology, Department of Environmental Science and Engineering, University of Science and Technology of China, Hefei, 230026 People’s Republic of China

**Keywords:** Nanomotor, MOFs, Selective biodegradation, Emerging contaminants, Water remediation

## Abstract

Catalytically powered micro-/nanomotors have become a compelling alternative to conventional catalysts for active and efficient removal of environmental pollutants in water remediation. We developed a novel biocatalytic nanomotor system by encapsulating catalase and peroxidase enzymes into metal–organic frameworks (MOFs), demonstrating exceptional speed and facilitated motion-induced convection and mass transfer. Leveraging a synergistic structural etching and surface engineering strategy using tannic acid (TA), we create a tailored microenvironment of the MOF’s framework with charge-selective and nanoconfinement properties. Both experimental and simulation results indicate that microenvironment modulation of MOF matrix could act in synergy with the encapsulated enzymes and significantly improve efficiency and selectivity in removing charged pollutants. Surface engineering of TA selectively preconcentrates target contaminants by modulating the MOF shell's surface charge, while etching-induced voids facilitate rapid mass transfer to the enzyme active sites. Finally, we also validated the applicability of these nanomotors in the transformative removal of pollutants from the aqueous phase into polymeric products via an enzyme-mediated polymerization pathway. This biocatalytic nanomotor system provides a promising water remediation paradigm for reducing carbon emissions and recycling chemical energy from emerging contaminants.

**Supplementary Information:**

The online version contains supplementary material available at 10.1007/s40820-025-02064-w.

## Highlights

- Biocatalytic metal–organic framework nanomotors were engineered with tunable microenvironment through a synergistic etching and surface engineering strategy.
- Enhanced catalytic efficiency and selectivity for dye decontamination were achieved through charge-based enrichment and nanoconfinement effects.
- Exceptional performance in water remediation of emerging contaminants, e.g., ~ 98% bisphenol A removal, in 2 min was achieved via enzymatic transformation into recoverable products.

**Supplementary Information:**

The online version contains supplementary material available at 10.1007/s40820-025-02064-w.

## Graphical abstract

**Figure:**
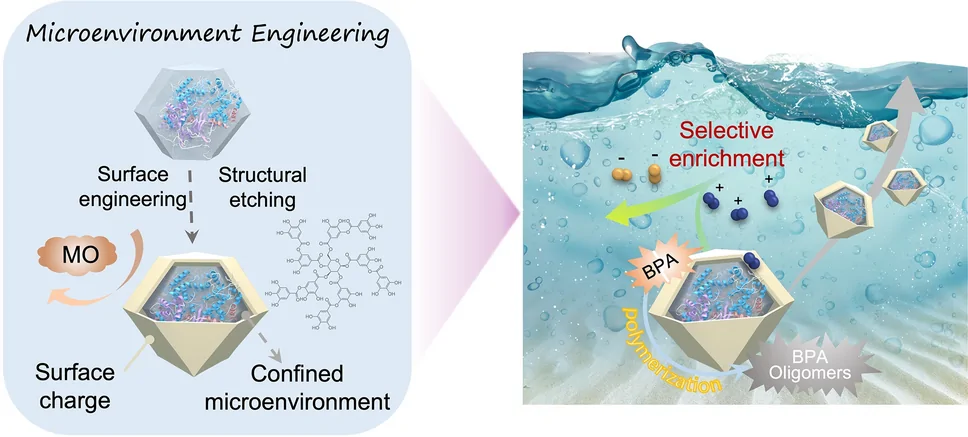

## Introduction

Water pollution poses a critical global challenge, necessitating the development of advanced technologies for efficient remediation [1, 2]. Self-propelled micro- and nanomotors offer significant advantages over passive nanoparticles in water treatment by enhancing mass transfer and enabling efficient micro-mixing of contaminants, which passive diffusion-based systems cannot achieve [3–5]. However, most current self-propelled systems rely on metallic micromotors [6, 7] and energy- or chemical-intensive advanced oxidation processes to achieve complete mineralization of organic pollutants, resulting in high CO_2_ emissions and unorganized release of chemical energy [8, 9]. In contrast, the desirable polymerization offers a greener alternative by avoiding the need for the total decomposition of the carbon skeletons of contaminants [10, 11], instead facilitating chemical energy recovery from organic wastes with lower chemical and energy inputs [12, 13]. A new paradigm of nanomotor systems for green remediation with mild oxidation, high selectivity, and low carbon footprint is therefore urgently desired.

In recent years, natural catalysts such as enzymes have sparked extensive interest as promising alternatives for driving micro-/nanomotor systems with high efficiency [14, 15]. Notably, these biocatalysts can catalyze the polymerization of phenolic compounds, presenting an innovative strategy for energy harvesting from toxic micropollutants by transforming them into polymeric products that are easier to recover [16, 17]. The intrinsically porous structure and adaptable surface functionality of metal–organic frameworks nanoparticles (MOFs) make them ideal hosts for developing biocatalytic nanomotors with enhanced catalytic activity [18, 19], versatility [14, 20], and stability [21], enabling applications in water remediation of dye [22], heavy metal [22], and PFOA [23]. In contrast to the conventional use of metal–organic frameworks (MOFs) as protective scaffolds or adsorptive materials [24, 25], our study focuses on tailoring the surface functionality of MOFs to modulate the microenvironment surrounding encapsulated enzymes, thereby enhancing selectivity toward specific target pollutants. Among various MOFs, ZIF-8 was selected because it can be synthesized under mild, enzyme-compatible conditions with high encapsulation efficiency and activity preservation, and its framework is readily engineerable for tailored structural and interfacial properties [26, 27]. While previous studies have primarily focused on improving reaction kinetics in catalytic oxidation, our approach differs by actively shifting the oxidation pathway from degradation to polymerization, enabling phenolic pollutants to be transformed into recoverable polymeric products, thus offering a more selective and sustainable solution for water decontamination.

**Fig. 1:**
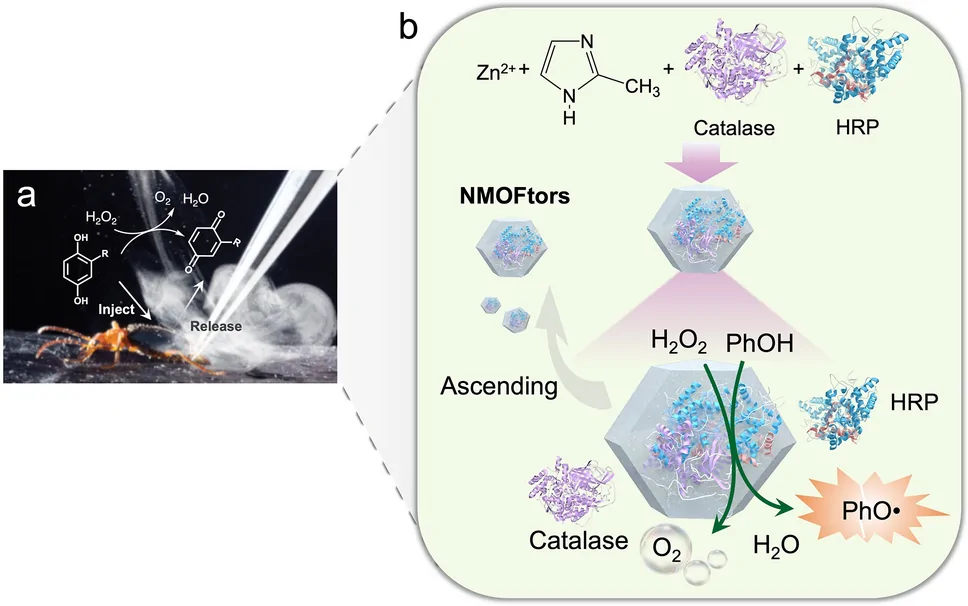
**a** Chemical reactions inside bombardier beetle chambers containing peroxidase and catalase enzymes: production of oxygen and catalytic oxidation of hydroquinone with H_2_O_2_ as the fuel[33]. **b** Schematic representation of the multistep process used to synthesize the biomimetic enzyme@ZIF-8 NMOFtors

The natural reaction chamber of bombardier beetles, which utilizes peroxidase and catalase enzymes with H_2_O_2_ as fuel to generate propulsion while oxidizing hydroquinone, served as inspiration for this study [28]. By encapsulating catalase (CAT) and horseradish peroxidase (HRP) within a nanoporous ZIF-8 shell, we developed a self-propelled biomimetic MOF-based nanomotor system (NMOFtors) for catalytic decontamination of targeted pollutants in aquatic environments (Fig. 1). The dual-enzyme design leverages the distinct catalytic roles of catalase and horseradish peroxidase (HRP). Catalase, with higher affinity for H_2_O_2_, rapidly decomposes excess peroxide into O_2_ and H_2_O, providing propulsion and maintaining a safe H_2_O_2_ level. HRP, in contrast, has lower affinity for H_2_O_2_ but higher affinity for phenolic substrates and catalyzes their oxidation in the presence of moderate H_2_O_2_ [29, 30]. This mechanistic divergence minimizes direct competition between pollutants and H_2_O_2_, while also preventing HRP inactivation at high H_2_O_2_ concentrations [31, 32].

This work introduces a synergistic etching and surface engineering strategy using tannic acid (TA) for rational modulation over the microenvironment of enzymes within MOF hosts. Demonstrated experimentally and by simulation, the tunable NMOFtor system achieved both high catalytic activity and selectivity toward harmful dyes, as a result of the synergistic contributions from the charge-based interactions and reactant enrichment under nanoconfinement. Furthermore, bisphenol A, a representative emerging aromatic contaminant, was employed as the model target to validate the robustness of this system, and the unique transformation pathway was elucidated by product analysis using the LC–MS/MS technique. This study provides valuable inspiration for the rational design and engineering of nanomotor systems toward high selectivity and sustainable performance in water decontamination.

## Experimental Section

### Materials

Zinc nitrate hexahydrate (99%), 2-methylimidazole (99%), tannic acid (TA, AR), catalase from bovine liver (CAT, 2000–5000 units mg^−1^), horseradish peroxidase (HRP, ≥ 250 units mg^−1^), methylene blue (MB, AR), methyl orange (MO, AR), bisphenol A (BPA, ≥ 99.0%), pyrogallol (AR), and fluorescein isothiocyanate (FITC, ≥ 97.5% HPLC) were purchased from Sigma-Aldrich, Australia. Humic acid (HA) as a model for natural organic matter (NOM) was supplied from Sigma-Aldrich. Illustra NAP-25 columns (17-0852-02, GE Healthcare) were used for the separation of labeled enzymes. UHPLC-MS/MS used acetonitrile and ammonium acetate were purchased from Merck (Australia) and of either LC–MS grade or ≥ 99.9% purity. Alexa Fluor 350 NHS ester (AF350, 95%) was purchased from Thermo Fisher Scientific (Australia). H_2_O_2_ content assay kit was purchased from Solarbio. All other reagents were purchased from Sigma-Aldrich (Australia) and used without further modification.

### Synthesis of Biocatalytic Enzyme@ZIF-8 NMOFtors

The synthesis of enzyme@ZIF-8 followed a previous report by our group with slight modifications. Briefly, 0.1 mL of prepared CAT solution (5 mg mL^−1^) and 0.1 mL of HRP solution (5 mg mL^−1^) were mixed with 0.8 mL of 2-methylimidazole solution (860 mM). Then, 0.2 mL of zinc nitrate solution (45 mM) was then added quickly followed by continuous stirring for 1 h. The obtained particles were collected by centrifugation (Eppendorf Centrifuge 5418) at 5000 rpm for 2 min. The particles were washed with Milli-Q water and ethanol three times and resuspended in 0.1 mL Milli-Q water.

### Surface Engineering and Controlled Etching

In a typical synthesis, the obtained enzyme@ZIF-8 polyhedrons were dispersed in 0.2 mL Milli-Q water and subsequently mixed with 0.4 mL tannic acid solution to obtain final TA concentrations of 2 − 10 g L^−1^. The mixture was vortexed and aged for 1–3 min at room temperature. The resultant TA-enzyme@ZIF-8 polyhedrons were collected by centrifugation and washed immediately with water and ethanol three times to remove the residual TA. The resulting nanoparticles were then resuspended in 0.1 mL of Milli-Q water for further studies.

### Selectivity and Catalytic Performance Test

The catalytic decontamination of methylene blue (MB) and methyl orange (MO) by the enzyme@ZIF-8 NMOFtors and the TA-engineered enzyme@ZIF-8 NMOFtors (TA-NMOFtors) was evaluated using UV–Vis spectroscopy by measuring the decrease of the absorbance of the respective absorption band over time (ε_660_ = 2.99 × 10^4^ M^−1^ cm^−1^ and ε_456_ = 1.19 × 10^4^ M^−1^ cm^−1^, respectively). The reactions were performed at room temperature with 50 μL NMOFtors (virgin and TA-engineered), 0.15% H_2_O_2_, and dye solution with increasing concentrations in a total volume of 2 mL. The decontamination efficacy of adsorptive enzyme@ZIF-8 NMOFtors and TA-NMOFtors system was also evaluated with the same amount used in their oxidative counterparts. The reaction rates were obtained by calculation of the slope of the respective absorbance over reaction time.

### BPA Decontamination Experiments

Remediation experiments were conducted in glass vials containing BPA solutions at concentrations ranging from 0.2 to 100 μM. A calculated volume of enzyme@ZIF-8 nanomotors was gently introduced to the bottom of each vial, achieving a final concentration of 0.1–0.3 g L^−1^. After the adsorption equilibrium of phenolic pollutants was reached, the motors were activated by adding hydrogen peroxide (0.15 wt%) as fuel solution and propelled through the BPA-contaminated solution. The impact of environmental factors (e.g., pH, ionic strength, organic matter, temperature) was investigated at a pH range of 4.5–10, 2–10 mM chloride (Cl^−^) and bicarbonate (HCO_3_^−^), 2–10 mg L^−1^ NOM, 25–60 °C. A sample of 0.25 mL was withdrawn at different time intervals, filtered using a 0.22-μm Millex-GP syringe filter before analysis. The concentration of BPA pollutants was quantified by a high-performance triple-stage quadrupole HPLC/MS Mass Spectrometer (Thermo TSQ Vantage) at electrospray ionization (ESI) mode. The LC mobile phase was a mixture of acetonitrile/2 mM ammonium acetate (70/30 v/v). The mass spectrometer was operated in the negative ion mode with a spray voltage of − 3500 V, over a scan range of m/z 50–500.

## Results and Discussion

### Preparation and Characterization of Enzyme-Encapsulated MOF Nanomotors

**Fig. 2:**
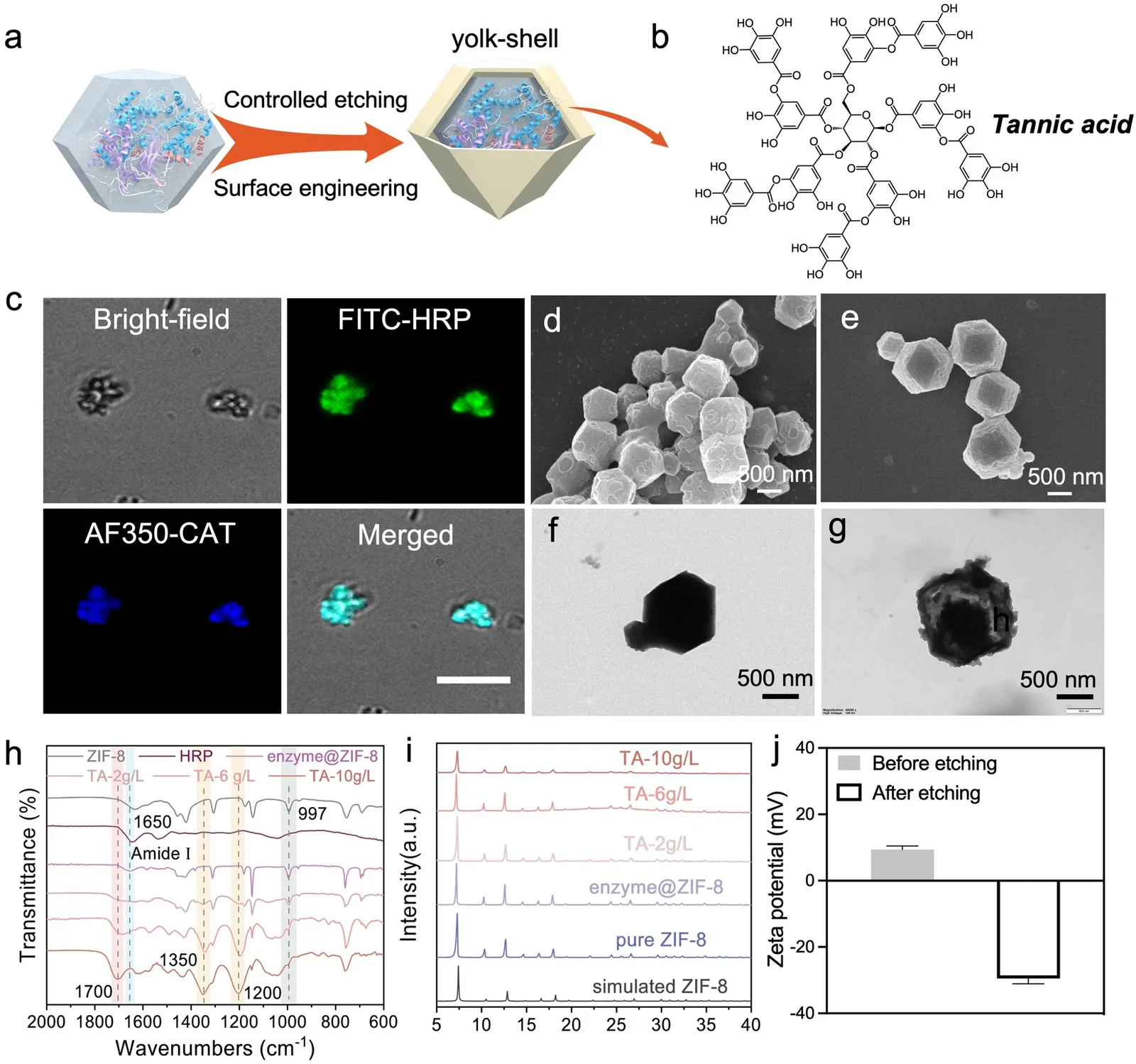
**a** Schematic illustration showing the surface engineering of enzyme@ZIF-8 and the etching process to engineer voids inside. **b** Molecular structures of tannic acid. **c** CLSM and bright field microscopy images of TA-enzyme@ZIF-8. CAT and HRP were labeled with AF350 and FITC, respectively (scale bar is 5 μm). SEM and TEM images of enzyme@ZIF-8 **d** and **f** before and **e** and **g** after TA treatment (6 g L^−1^). **h** FTIR spectra of HRP, pure ZIF-8, enzyme@ZIF-8, and TA-enzyme@ZIF-8 after etching with varying TA concentrations (2, 6, and 10 g L^−1^). **i** PXRD patterns of simulated ZIF-8, pure ZIF-8 and the enzyme@ZIF-8 before and after treatment with varying TA concentrations (2, 6, and 10 g L^−1^). **j** ζ-potential of enzyme@ZIF-8 before and after TA treatment

The preparation procedure of enzyme@ZIF-8 and its surface engineering process is expounded systematically in Figs. 1 and 2a. Two enzymes, CAT and HRP, were first entrapped within the nanoporous ZIF-8 framework composed of Zn^2+^ linked by the 2-methylimidazole ligand, forming enzyme@ZIF-8 NMOFs. Previous research has proven that enzymes facilitate the nucleation of ZIF-8 by increasing the local concentration of building blocks (both Zn^2+^ ions and organic ligands) around the biomacromolecules [34]. The dispersed enzyme@ZIF-8 crystals were subsequently subjected to TA treatment for surface engineering and controlled etching. The spatial confinement of the two enzymes within the MOFs was further confirmed by confocal laser scanning microscopy (CLSM) using FITC-labeled HRP and AF350-labeled catalase (Figs. 2c and S1). Green fluorescence of FITC-labeled HRP was observed upon excitation of MOFs at 483 nm. Similarly, excitation of the MOFs at 346 nm yielded blue fluorescence from AF350-labeled CAT. The merged images confirmed homogeneous co-localization of the CAT and HRP labeled with different fluorophores, throughout the entire ZIF-8 nanocrystals, with no changes in spatial distribution after TA etching. The loading efficiency of enzymes within ZIF-8 was determined by fluorescence spectrophotometry (Fig. S2). The results showed that 94% CAT and 83% HRP were immobilized in the nanomotor.

Brunauer–Emmett–Teller (BET) analysis (Fig. S3 and Table S1) demonstrated the generation of mesoporosity after TA etching despite partial reduction in the microporous surface area, as evidenced by the appearance of a pronounced adsorption–desorption hysteresis loop in the N_2_ isotherm and a shift in the pore size distribution. Scanning electron microscopy (SEM) images indicate that the obtained biocomposites after TA treatment retained the same morphology as the initial enzyme@ZIF-8 nanoparticles, showing a classical rhombic dodecahedron morphology with a particle size of 0.7–1.0 μm (Fig. 2d, e). Energy-dispersive spectrum (EDS) mapping (Figs. S4 and S5) confirmed the co-distribution of Zn and O (from 2-MeIM and enzyme) in nanoparticles. X-ray photoelectron spectroscopy (XPS) analyses in Fig. S6 confirmed enzyme encapsulation by detecting amide-N (400.26 eV) and O 1*s* signals, species theoretically absent in the pure ZIF-8 framework. Upon TA etching, the O 1*s* spectrum showed a marked increase in the C–O peak proportion (533.21 eV), and the C 1*s* carbonyl peak shifted to 288.65 eV (ester), verifying the TA coating. Notably, N 1*s* peaks shifted to higher binding energies (+ 0.60–0.76 eV), attributed to nitrogen protonation by TA’s phenolic groups. Transmission electron microscopy (TEM) imaging revealed that the polyhedral external morphology of the initial enzyme@ZIF-8 nanoparticles is largely retained after TA etching. However, an increase in internal low-contrast regions can be observed with rising TA concentration, indicative of the formation of internal voids. This suggests a progressive transformation from dense solid particles to yolk–shell structures (Fig. 2f, g), which facilitates enhanced substrate diffusion and increased accessibility of catalytic sites. Notably, at a lower TA concentration (2 g L^−1^, Fig. S7a), the particles exhibited a relatively uniform and dense structure with minimal internal voids, indicating insufficient etching. At a higher concentration (10 g L^−1^, Fig. S7b), the particles displayed pronounced hollow interiors, suggesting aggressive core dissolution due to more extensive etching.

Fourier transform infrared spectroscopy (FTIR) spectroscopy in Fig. 2h further supported this by showing an increased signal intensity near 1700 cm^−1^ with increasing TA dosage. This region corresponds to the C=O stretching in TA, which overlaps with the enzyme’s amide I band (1700–1610 cm^−1^) [35, 36]. Additionally, an increase in signal intensity around 1350–1150 cm^−1^ was observed, corresponding to C**–**O stretching. The spectral enhancement and overlap suggest substantial surface adsorption of TA at higher concentrations, which may obscure protein-related signals and weaken the characteristic ZIF-8 signal at 997 cm^−1^, indicating increasing TA accumulation on the MOF surface. Powder X-ray diffraction (PXRD) analysis (Fig. 2i) showed that the characteristic diffraction peaks of ZIF-8 (2θ = 7.18°, 10.4°, 12.6°, 17.9°) were preserved under 2 and 6 g L^−1^ TA etching, indicating retention of crystallinity. However, at 10 g L^−1^, a reduction in peak intensity and mild broadening were observed. This can be attributed to both partial disruption of the internal framework due to over-etching and, more importantly, to thick amorphous TA coating attenuating the crystal signal, consistent with the FTIR observations. Together, these results demonstrated the concentration-dependent structural evolution of enzyme@ZIF-8 upon TA treatment and provide a strategy for controllable MOF microenvironment engineering. As revealed in Fig. 2j, a significant decrease in the zeta potential of enzyme@ZIF-8 nanoparticles was observed after TA etching, resulting in the reverse of the surface charge from positive to negative. Contact angle measurements in Fig. S8 indicated that TA modification increased the hydrophilicity of the ZIF-8 surface, with the contact angle decreasing from 65.8° (pristine enzyme@ZIF-8) to 55.5° (TA-modified enzyme@ZIF-8).

Based on the above observations and previous reports [37, 38], we proposed that TA regulates the microenvironment of enzyme@ZIF-8 through a synergistic etching and surface engineering mechanism. The etching process begins with the adsorption of TA molecules onto the hydrophobic surface of ZIF-8, increasing surface hydrophilicity and facilitating the infiltration of water molecules and protons into the framework. Since TA is a weak organic acid, the proton is gradually released and continuously consumed during the etching process. This results in a mild, self-limiting etching process, with the pH gradually increasing and eventually neutralizing the etching environment (pH ~ 8), naturally terminating the reaction (Fig. S9). Notably, the pH of the reaction mixture after TA addition was measured at 6.52, which closely aligns with the natural pH of the enzyme solutions (CAT: 6.68; HRP: 7.10 in DI water), providing a mild etching environment that preserves enzyme conformation and avoids inactivation. In addition, owing to its large molecular size (average MW ≈ 1700 Da) [39], TA cannot penetrate the internal micropores of ZIF-8 and therefore serves as a surface-bound protective layer that partially shields the outer shell while allowing localized etching within the core. This leads to the formation of yolk–shell-like MOF structures with preserved polyhedral morphology and crystallinity, as confirmed by TEM and PXRD results. Additionally, FTIR analysis in Fig. 2h showed increased surface-bound TA signals, further supporting the protective coating effect.

### Enzymatic Performance and Motility Behavior of NMOFtors

**Fig. 3:**
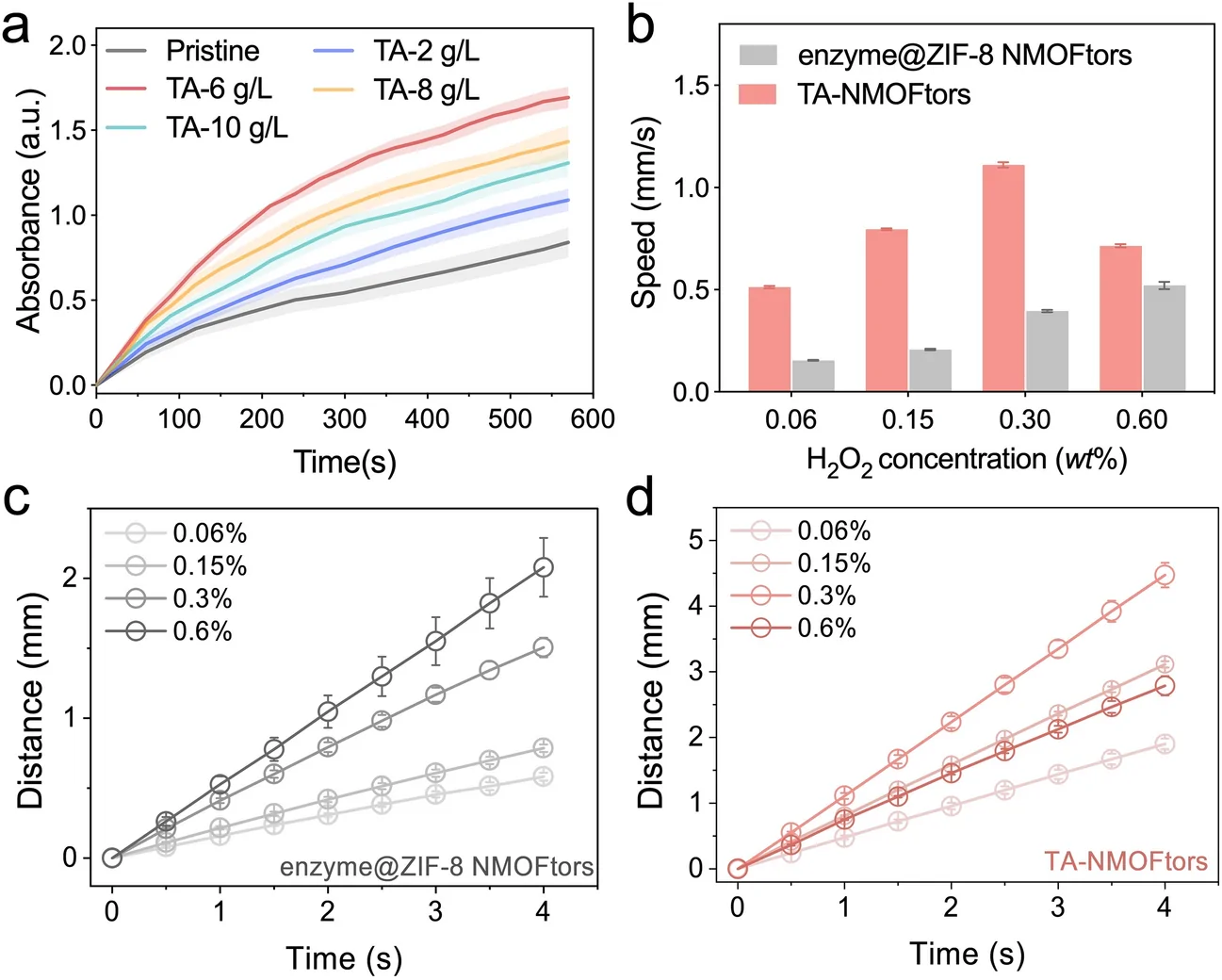
**a** Time-dependent enzymatic kinetics of enzyme@ZIF-8 NMOFtors after etching with varying TA concentrations (2, 6, 8, and 10 g L^−1^), showing optimal performance at 6 g L^−1^. **b** Ascent velocity of the NMOFtors at different H_2_O_2_ concentrations. Error bars represent the standard error of the mean calculated from the instantaneous velocities of three individually tracked particles, as extracted from video-based trajectory analysis. **c** Moving distance of enzyme@ZIF-8 NMOFtors, and **d** TA-NMOFtors with different concentrations of hydrogen peroxide ranging from 0.06% to 0.6%

To investigate the etching effect of TA on the microenvironment of enzyme@ZIF-8 and its impact on enzymatic performance, we evaluated the enzyme activity of NMOFtors treated with varying concentrations of TA (2, 6, 8, and 10 g L^−1^), using pyrogallol as the substrate and monitoring the formation of the yellow-colored purpurogallin at 420 nm by UV–Vis spectroscopy. Among the tested conditions, the sample etched with 6 g L^−1^ TA for 3 min exhibited the highest catalytic activity, showing a 2.1-fold increase compared to the pristine enzyme@ZIF-8. Further decreasing the TA concentration from 6 to 2 g L^−1^ or increasing it to 10 g L^−1^ led to a compromised enhancement in enzymatic activity (Fig. 3a). When enzyme@ZIF-8 is subject to a lower TA concentration or too short etching duration, incomplete etching may occur, which restricts the diffusion of substrates and intermediates, thereby limiting further enhancement of enzyme activity. In contrast, a prolonged etching time or higher TA concentration may result in excessive TA deposition on the ZIF-8 surface, as supported by FTIR analysis, which can potentially block external micropores and reduce substrate accessibility (Figs. 3a and S10). Based on the above analysis, a TA concentration of 6 g L^−1^ for 3 min was identified as the optimal condition. For long-term stability, TA-enzyme@ZIF-8 retained ~ 76% of its initial catalytic activity after 10 reaction cycles, whereas free HRP lost all activity after one cycle (Fig. S11). Additionally, the TA-modified nanomotors exhibit less than 10% loss of enzymatic activity after two weeks at 4 °C, with preserved MOF crystalline structure confirmed by the PXRD (Fig. S12), demonstrating the durability of TA modification.

In our system, hydrogen peroxide (H_2_O_2_) serves as both an oxidizing agent and chemical fuel, driving nanomotor propulsion and catalytic reactions. Upon exposure to H_2_O_2_, the NMOFtors exhibited immediate cyclic vertical motion, accompanied by rapid oxygen bubble release (Video S1), which was recorded and analyzed by optical microscopy (Videos S2–S3). To correlate propulsion performance with catalytic activity, gradient experiments were conducted over a range of H_2_O_2_ concentrations (0.06–0.6%, v/v). As shown in Fig. 3b–d, the velocity of enzyme@ZIF-8 NMOFtors increased with fuel concentration, reaching 523 ± 18 μm s^−1^ at 0.6%. In contrast, the TA-NMOFtors exhibited significantly enhanced propulsion compared to most previously reported micro/nanomotors, achieving a maximum velocity of 1113 ± 12 μm s^−1^ at 0.3%, before decreasing to 720 ± 7 μm s^−1^ at 0.6%. Dissolved oxygen (DO) measurements in Fig. S13 revealed that TA-NMOFtors exhibited a peak O_2_ generation rate of ~ 18.2 μmol min^−1^ mg^−1^ at 0.3% H_2_O_2_, which decreased to ~ 12.0 μmol min^−1^ mg^−1^ at 0.6%, paralleling the observed propulsion trend. In contrast, pristine NMOFtors showed a steady increase in both speed and O_2_ generation (from 4.5 to 10.7 μmol min^−1^ mg^−1^), albeit with lower overall performance, indicating a reaction-limited regime without evident saturation.

These observations were further validated by kinetic measurements of H_2_O_2_ decomposition using a titanium sulfate colorimetric assay (Figs. S14 and S15). The TA-NMOFtors showed a higher observed rate constant (k_obs_ = 0.0229 s^−1^) than pristine NMOFtors (k_obs_ = 0.0108 s^−1^), confirming improved catalytic activity and H_2_O_2_ utilization efficiency. This enhancement is primarily attributed to TA-induced mesoporous apertures and a hydrophilic microenvironment, which favorably facilitate the rapid diffusion of substrate (H_2_O_2_) and product (O_2_), thereby promoting efficient catalysis and propulsion. Taking into account the above findings as well as the importance of minimizing potential chemical toxicity for environmental applications, 0.15% H_2_O_2_ was chosen as the optimal working concentration for the following decontamination tests.

### Microenvironmental Modulation on Catalytic Selectivity and Efficiency

**Fig. 4:**
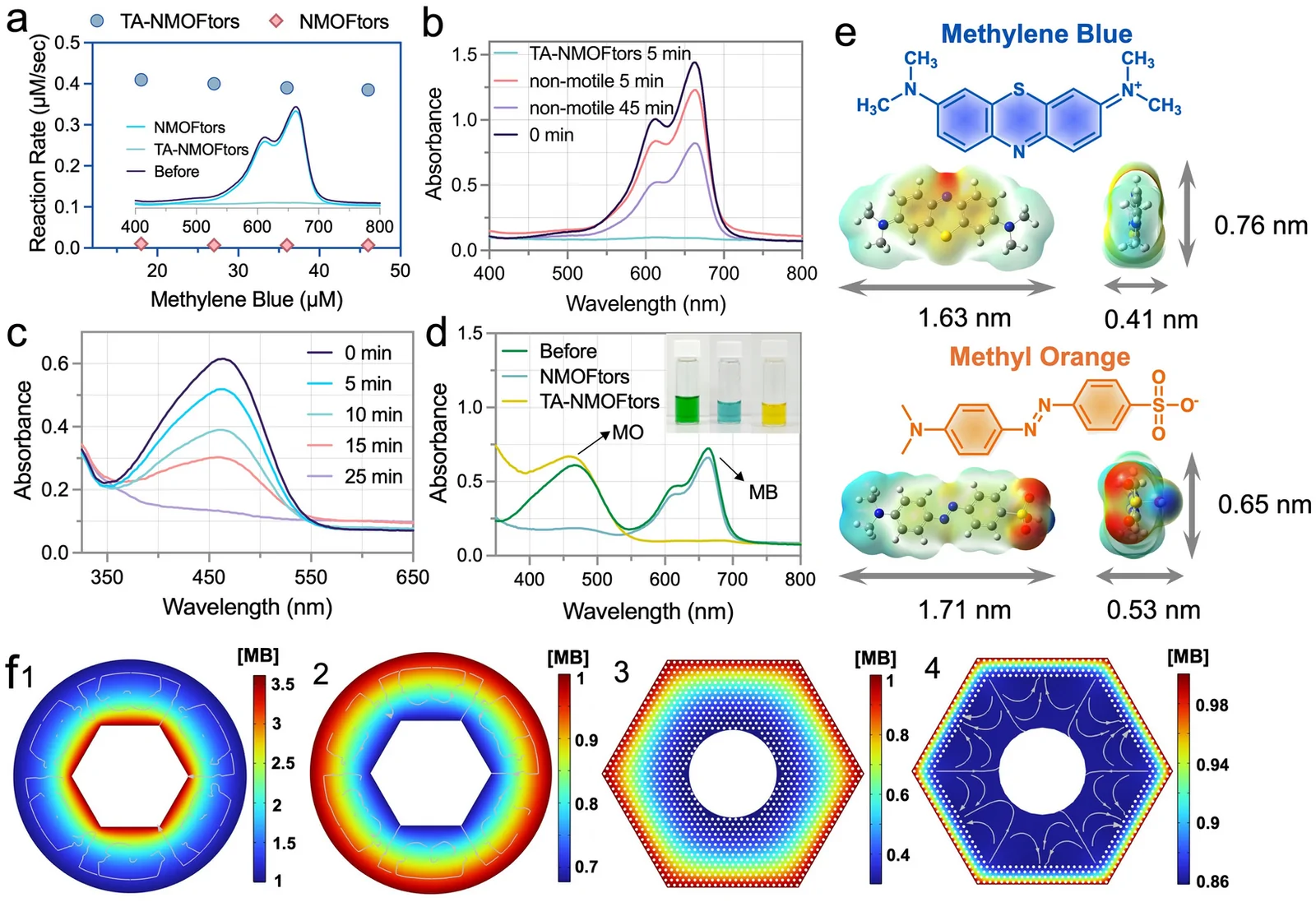
**a** Reaction rate of the decontamination process of MB by enzyme@ZIF-8 NMOFtors (red diamonds) and TA-NMOFtors (blue circles); **b** UV–Vis absorbance spectra of MB solutions before and after decontamination by motile and non-motile TA-NMOFtors; **c** time evolution of the typical UV–Vis spectra during MO decontamination by enzyme@ZIF-8 NMOFtors; **d** UV–Vis spectra and optical images of MB/MO mixtures before and after incubation with enzyme@ZIF-8 NMOFtors and TA-NMOFtors; **e** optimized molecular structure of MB (positive) and MO (negative) and their electrostatic potentials (ESP). Red and blue regions represent negative and positive ESPs, respectively; **f** simulated concentrations and distributions of local MB on the surface of TA-NMOFtors (f_1_) and enzyme@ZIF-8 NMOFtors (f_2_), and its diffusion process inside solid enzyme@ZIF-8 NMOFtors (f_3_) and TA-NMOFtors (f_4_)

On the basis of the above results and previous work [37, 38], we propose that TA engineers MOFs via synergistic surface functionalization and void etching. Herein, our idea is to investigate how the surface functionality and hollow structure would affect the adsorption selectivity of the MOF matrix and enzymatic activity and thus may lead to an enhanced dye decontamination performance with tunable selectivity. Model harmful dyes with similar molecular diameters but different electrostatic charges were thus employed to verify this hypothesis: methylene blue (MB, cationic), and methyl orange (MO, anionic), as shown in Fig. 4. The separation of their mixtures has been reported with various types of MOFs [24, 40], among which ZIF-8 particles exhibit selective adsorption of anionic MO over cationic MB due to electrostatic interactions [41]. In this study, the catalytic activity toward the decontamination of MB and MO by the pristine enzyme@ZIF-8 NMOFtors and the TA-NMOFtors was thus investigated.

Figure 4 shows the UV–Vis absorbance spectra of the MB and MO removal experiments when NMOFtors were navigated in a contaminated solution for different durations. For the oxidation of the positively charged dye (MB, Fig. 4a), the reaction rate observed with enzyme@ZIF-8 NMOFtors was almost negligible (k = 0.0068 μM s^−1^), which can be explained by the electrostatic repulsive interaction between MB and the ZIF-8 scaffold. In contrast, TA-NMOFtors encapsulating HRP achieved a markedly higher reaction rate (k = 0.39 μM s^−1^), which can be attributed to microenvironmental effects induced by TA engineering. Specifically, the electrostatic attraction between MB and the TA-coated MOF matrix may strongly favor the preconcentration of MB in the vicinity of HRP. The control non-motile TA-NMOFtors showed a lower reaction rate (k = 0.023) with only 14.6% removal efficiency within 5 min of reaction (Fig. 4b). For comparison, the active TA-CAT@ZIF-8 nanomotors (TA-CAT@NMOFtors) possessed appreciable removal efficiency of approximately 81.7% in the presence of 0.15% hydrogen peroxide, thus confirming the promotive effect of propelled motion on the performance of NMOFtors (Fig. S16). The oxidative TA-NMOFtors showed a further 17% enhancement in removal efficiency to 98.7% within 5 min of incubation (Fig. 4b). The results confirmed that the surface engineering of the MOF matrix with TA contributed to highly efficient dye decontamination efficiency by providing a charge-based selective preconcentration of reactants to facilitate HRP oxidative catalytic activity.

Similar experiments were carried out with negatively charged dye (MO, Fig. 4e). The TA-NMOFtors showed relatively negligible MO removal (k = 0.007 μM s^−1^) when compared to original enzyme@ZIF-8 NMOFtors (Fig. S17). This can be explained by electrostatic repulsion interactions between MO and TA-NMOFtors with highly negative surface charge, which hinders the adsorption of anionic MO. In the presence of 0.15% H_2_O_2_, the adsorptive CAT@ZIF-8 nanomotors (CAT@NMOFtors) showed 30.4% removal efficiency within 25 min (k = 0.012 μM s^−1^, Fig. S18), while oxidative enzyme@ZIF-8 NMOFtors showed an enhanced reaction rate (k = 0.046 μM s^−1^) and almost complete MO removal during the same period (Fig. 4c). For comparison, without using H_2_O_2_, non-motile enzyme@ZIF-8 NMOFtors exhibited very limited removal toward MO even after 45 min (Fig. S19). Collectively, the controlled etching of MOF with TA contributed to the motors’ overall enhancement in catalytic efficiency, which can be explained by the constructed hierarchically porous structure accelerating the diffusion rate of reactants and spatial confinement that facilitates accommodated enzymatic reactions.

Figure 4d displays the optical and UV–Vis spectra of the MB/MO mixtures before and after incubation with enzyme@ZIF-8 NMOFtors and their TA-treated counterparts, respectively. The original green color, which reflected the simultaneous presence of both dye species, turned to blue with enzyme@ZIF-8 NMOFtors and yellow with TA-NMOFtors, revealing the preferential affinity toward anionic MO and cationic MB, respectively. TA-NMOFtors exhibited a high separation factor of approximately 73 for MB and MO within 5 min. These results showed a charge-selective removal performance consistent with the results of the single-component decontamination experiments.

Finite element simulation was performed to illustrate surface enrichment of different species on the surface of enzyme@ZIF-8 NMOFtors and TA-NMOFtors and to further analyze their diffusion process. The negatively charged surface selectively enriches oppositely charged dye species via electrostatic attraction, resulting in a higher local MB concentration on TA-NMOFtors (Fig. 4f1), whereas solutes with the same charge showed lower surface concentration due to electrostatic repulsion (Figs. 4f2 and S19). In solid structures, diffusion followed a long path with substantial adsorption losses along pore walls, leaving most pollutants near the MOF pores and limiting the accessibility to enzyme sites (Figs. 4f3 and S20). By contrast, TA etching produced a hollow structure with a thinner, porous shell, shortening diffusion pathways and allowing target molecules to traverse the shell and accumulate in the cavity around the enzymes (Figs. 4f4 and S21). The simulation results, corroborated by experiments, confirm that TA-induced synergistic etching and surface engineering contributed to an overall enhanced enzymatic activity and charge-based selectivity by optimizing mass transfer contact with the encapsulated enzymes and electrostatic affinity with target species.

### Application in Water Remediation of Emerging Contaminant

Apart from harmful dyes, emerging contaminants (ECs) prevalent in aquatic environments, such as endocrine-disrupting chemicals, pose significant risks to human health and remain challenging to remove efficiently using conventional water treatment processes. Building on the strong catalytic performance of biocatalytic NMOFtors in removing charged dyes, we further explored their applicability to the decontamination of neutral pollutants, using bisphenol A (BPA) as a representative target (Fig. S22a).

As shown in Fig. S22b, active CAT@NMOFtors showed improved BPA removal efficiency to over 80% within 2 min, outperforming non-motile nanomotors (w/o H_2_O_2_) due to enhanced mass transfer by self-propulsion, while oxidative enzyme@ZIF-8 NMOFtors further elevated removal efficiency to 98%. For TA-modified NMOFtors, the adsorptive TA-CAT@NMOFtors showed a reduced efficiency of 74%, likely due to decreased surface area and weakened *π*–*π* interaction after TA etching. Nonetheless, oxidative TA-NMOFtors still exhibited an apparent kinetic rate constant of 1.27 min^−1^, comparable to enzyme@ZIF-8 NMOFtors (*k*_obs_ = 1.51 min^−1^). This can be explained by the hollow structure in TA-NMOFtors, which accelerates the transport of small reactants and intermediates to the vicinity of HRP active sites, thus enhancing biocatalytic oxidation efficiency. The oxidative nanomotors also demonstrated robust performance across a wide BPA concentration range of 0.2–100 μM, demonstrating their applicability under environmentally relevant conditions (Fig. S23). Notably, optimizing the dosage of TA-NMOFtors within the range of 0.2–0.3 g L^−1^ significantly enhanced BPA removal performance, achieving 94.5–99% BPA removal (Fig. S24). An optimal dosage of 0.25 g L^−1^ was identified, providing high removal efficiency while minimizing material usage.

To evaluate the practical applicability of the NMOFtors in real-world water treatment conditions, we systematically assessed the influence of environmental factors, including pH, ionic strength, and natural organic matter (NOM), as well as performance in real water samples. Our experimental results (Fig. S22c) demonstrated that TA-NMOFtors retained high activity across a broad pH range of 5–10, but showed reduced structural integrity and enzymatic activity under acidic conditions (pH < 5), delineating their applicable pH window and the limitations for use in strongly acidic wastewater scenarios. Although the presence of background ions (e.g., Cl^−^ and HCO_3_^−^) and NOM (up to 10 mg/L) caused slight inhibitory effects on BPA removal efficiency, the TA-NMOFtor system retained over 90% of its original removal efficiency (Fig. S22c), likely due to the size exclusion effect and surface electrostatic repulsion by negatively charged TA-NMOFtors (Figs. S25 and S26). Moreover, the TA-NMOFtor system retained 90.4% of its original activity at 60 °C, indicating robust resistance to temperature fluctuations typical in real water environments (Fig. S22d).

BPA decontamination tests in tap water and river water spiked with 20 μM BPA showed removal efficiency of 91.2% and 88.7% relative to the pure water benchmark, respectively, confirming system robustness in natural matrices (Fig. S22d and Table S2). These findings collectively underscore the exceptional environmental resilience of the NMOFtors and define their performance boundaries under realistic water treatment conditions. In addition, we also evaluated the possibility of recycling the NMOFtors while removing ECs, as displayed in Fig. S22d. The removal efficiency of adsorptive CAT@NMOFtors was not sustained after 5 cycles, declining to 60.5% of its original performance due to adsorption saturation. In contrast, for the oxidative counterparts, over 80% of the initial activity was well preserved for both TA-etched and unetched NMOFtors after 10 catalytic cycles of BPA decontamination. PXRD, SEM, and FTIR analyses show that enzyme@ZIF-8 NMOFtors maintain structural integrity after cycling (Figs. S11 and S27). Fluorescence-labeling and CLSM measurements indicate enzyme leakage of < 6% with strong fluorescence retained inside the particles, confirming that the activity loss stems from partial enzyme deactivation rather than enzyme escape from the MOF (Figs. S28 and S29). Compared with previously reported enzyme-based and MOF-based catalytic systems for water decontamination (Table S3), the biocatalytic NMOFtors system described in this study achieves markedly higher propulsion speed and rapid pollutant removal under low fuel concentration, while also maintaining excellent recyclability.

**Fig. 5:**
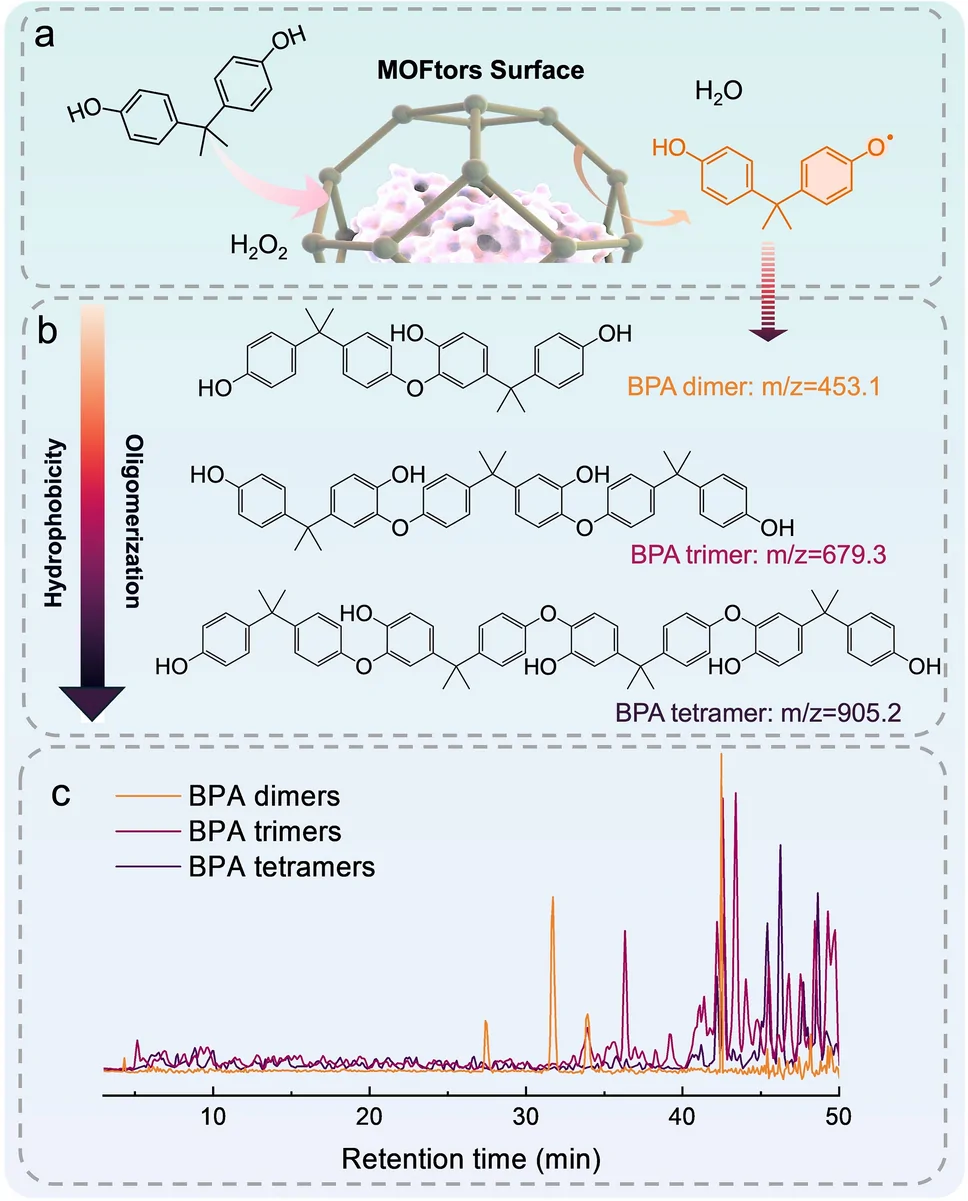
Proposed mechanism of BPA conversion mediated by biocatalytic NMOFtors: **a** formation of phenoxy radicals catalyzed by HRP within NMOFtors; **b** generation of BPA dimers, trimers, and tetramers with increased hydrophobicity; **c** extracted EIC of oligomeric products formed during repeated BPA treatment by biocatalytic NMOFtors. The peak intensities were normalized to enhance the visual comparison. Detailed MS data are provided in Figs. S30–S33

We employed UHPLC-MS/MS techniques to analyze extracted solid-phase products and elucidate the mechanism of phenolic contaminant removal by the NMOFtors system (Fig. 5, Supporting Information S1.10 and S1.11). HPLC/ESI–MS chromatograms and the fragmentation pattern of three main intermediates (Figs. S30–S33) revealed that BPA conversion mediated by biocatalytic NMOFtors produced large-molecular-weight products with m/z values of 453.1, 679.3, and 905.2, corresponding to BPA dimer (C_30_H_30_O_4_), trimer (C_45_H_44_O_6_), and tetramer (C_60_H_58_O_8_), respectively. Gradient elution with a mobile phase transitioning from water to acetonitrile facilitated the separation of transformed products, with hydrophobic compounds eluting later than hydrophilic ones. As indicated by the extracted ion chromatogram (EIC) results in Fig. 5c, the transformed products with a higher degree of oligomerization displayed increased hydrophobicity. The proposed mechanism involves HRP-catalyzed hydrogen abstraction, transforming small-molecule phenol contaminants into phenoxy radicals that couple and polymerize into high-degree oligomers with reduced estrogenic activity [42–44]. Due to their increased oligomerization degree and hydrophobicity, these oligomers bind to the MOF surface and can be readily removed from water with the process of NMOFtors separation by simple filtration [45]. Collectively, this nanomotor system involving enzyme-catalyzed oxidative coupling and polymerization reactions has potential utility as a water remediation technique for emerging contaminants removal by converting them into higher molecular weight polymers, which are typically of low solubility and can be readily separated from water via filtration.

## Conclusions

In conclusion, we constructed biocatalytic nanomotors by encapsulating dual enzymes within metal–organic frameworks for selective and catalytic decontamination of targeted contaminants in water. The entrapped catalase provided jet-like bubble propulsion, enabling MOF crystals to operate efficiently at low chemical fuel levels without external agitation. A dual microenvironment modulation strategy was established, employing tannic acid for synergistic etching and surface engineering to create a tailored microenvironment with optimized surface charge and nanoconfinement within the MOFs. This design significantly enhanced catalytic efficiency and selectivity for targeted dye decontamination by promoting charge-based reactant enrichment and improving mass transfer to enzyme active sites. Moreover, the developed biocatalytic nanomotors demonstrated exceptional recyclability and effectiveness in removing estrogenic phenolic contaminants through a natural transformation pathway. This work provides significant inspiration for designing advanced biocatalytic nanomotors and fine-tuning the multilevel microenvironment surrounding enzymes on a rational and predictive basis, offering a promising solution for targeted and sustainable water decontamination.

## Supplementary Information

Below is the link to the electronic supplementary material.

Supplementary file1 (DOCX 19432 KB)

Supplementary file2 (MP4 15189 KB)

Supplementary file3 (MP4 1249 KB)

Supplementary file4 (MP4 977 KB)
