# Supplementary material for: Microenvironment-Engineered Biocatalytic Metal–Organic Framework Nanomotors for Selective and Transformative Water Decontamination
Source: Nanomicro Lett. 2026 Jan 26;18:224. doi: 10.1007/s40820-025-02064-w (PMC12834862; doi:10.1007/s40820-025-02064-w)
Supplement: Supplementary file 1 — Supplementary file1 (DOCX 19432 KB) [file 40820_2025_2064_MOESM1_ESM.docx]

Supporting Information for

**Microenvironment-Engineered Biocatalytic** **Metal-Organic Framework Nanomotors for Selective and Transformative Water Decontamination**

Shu Xu ^1, 2^, Jueyi Xue^2^, Linyun Bao^4^, Joel Yong^2^, Ying Cao^3^, Jun Ma^3^, and Kang Liang^2,^ *

^1^ SCNU Environmental Research Institute, Guangdong Provincial Key Laboratory of Chemical Pollution and Environmental Safety & MOE Key Laboratory of Theoretical Chemistry of Environment, School of Environment, South China Normal University, University Town, Guangzhou 510006, P. R. China

^2^ School of Chemical Engineering and Graduate School of Biomedical Engineering, The University of New South Wales, Sydney, NSW 2052, Australia

^3^ State Key Laboratory of Urban Water Resource and Environment, School of Environment, Harbin Institute of Technology, Harbin 150090, P. R. China

^4^ State Key Laboratory of Advanced Environmental Technology, Department of Environmental Science and Engineering, University of Science and Technology of China, Hefei, P. R. China

*Corresponding author. E-mail: [kang.liang@unsw.edu.au](mailto:kang.liang@unsw.edu.au) (Kang Liang)

S1 Experimental Section

**S1.1 Materials**

Zinc nitrate hexahydrate, 2-methylimidazole, tannic acid (TA), catalase from bovine liver (CAT), horseradish peroxidase (HRP), methylene blue (MB), methyl orange (MO), bisphenol-A (BPA) and fluorescein isothiocyanate (FITC) were purchased from Sigma Aldrich Australia. UHPLC-MS/MS used acetonitrile and ammonium acetate were purchased from Merck (Australia) and of either LC-MS grade or > 99.9% purity. Alexa Fluor 350 NHS ester (AF350) was purchased from Thermo Fisher Scientific (Australia). All other reagents were purchased from Sigma Aldrich (Australia) and used without further modification.

**S1.2 Characterization**

Fluorescent microscope images were collected by Olympus IX53 inverted microscope equipped with sCMOS camera. Scanning electron microscope (SEM) characterization of samples was conducted on a FEI Nova Nano SEM 450 FE-SEM at an acceleration voltage of 10.0 kV. Transmission electron microscopy (TEM) images were taken using FEI Tecnai G2 F20. The crystallinity of enzyme@ZIF-8 motors was analyzed by PANalytical Xpert Materials Research diffractometer X-ray diffraction (XRD) system at 2θ from 5° to 40° with a 0.05° step size. The particle movement was recorded as a video using OneAttension Optical Tensiometer with High-speed camera modes, and the moving distance was calibrated with a 1 mm calibration slide (TS-M3/106021, 1DIV=0.01mm). ImageJ software was used to tract the particle motion curve that enabled the calculation of the particle velocity change from the video. ζ-potential studies and diameters of NOM were performed on a Malvern Instrument Zetasizer Nano ZS. Fourier transform infrared (FTIR) spectra were collected by a PerkinElmer Spectrum Two FT-IR Spectrometer. The UV−vis spectra were obtained using a multi-detection microplate reader (CLARIOstar Plus, BMG LABTECH). A Triple Stage Quadrupole LC/MS Mass Spectrometer (LC-MS/MS, TSQ Vantage, Thermo Scientific) was employed to measure the concentrations of emerging pollutants. Nitrogen adsorption–desorption isotherms were measured using a Micromeritics ASAP 2460 System at 77 K. The X-ray photoelectron spectra (XPS) was obtained on a Thermo Scientific ESCALAB 250Xi apparatus.

**S1.3 Enzyme encapsulation efficiency**

HRP and CAT were first labelled with FITC and AF350, respectively. Briefly, 5 µL of fluorescent dye (1 mg mL^–1^) was added into respective enzyme solution and mixed for 2 h on a shaking incubator under dark. The labelled enzymes were collected by filtration via NAP-25 column (GE Healthcare) to separate from the unreacted dye, and then encapsulated into ZIF-8 through above biomineralization method. [S1, S2] Fluorescent spectrophotometry was utilized to determine the amount of fluorescently labelled enzymes unencapsulated within ZIF-8 by comparison with their calibration curves. The supernatant after encapsulation was collected to determine the fluorescence intensity by a microplate reader [S3–S5].

For visualizing the spatial distribution of encapsulated enzymes, the raw enzymes were replaced by the dye-labeled ones to prepare the enzyme@ZIF-8 nanomotors. The distribution of dye-labeled enzymes in the ZIF-8 was surveyed by a confocal laser scanning microscope (Olympus IX53, Japan).

**S1.4 Biocatalytic activity assay**

The biocatalytic activity of enzyme@ZIF-8 NMOFtors and TA-NMOFtors was evaluated using pyrogallol and hydrogen peroxide as substrates. In a typical assay, 50 µL of NMOFtors suspension (5 mg mL^–1^ dispersed in deionized water), 50 µL of H_2_O_2_ solution (0.5% v/v, freshly prepared), and 100 µL of pyrogallol solution (50 mg mL^–1^ in deionized water, freshly prepared and protected from light) were added to 800 µL of Tris buffer (0.01 M, pH=7), giving a total reaction volume of 1.0 mL. The reaction mixture was gently vortexed, and the increase in absorbance at 420 nm, corresponding to the formation of purpurogallin, was monitored at 30 s intervals using a UV–Vis spectrophotometer. The relative enzymatic activity was calculated from the initial linear slope of the absorbance versus time curve.

**S1.5 Stability and recyclability test**

The recycling of NMOFtors was performed using 0.2 g/L of the TA-NMOFtors, 5 mg/ml pyrogallol, 2.5 mM H_2_O_2_ in 1 mL of Tris buffer (0.01 M) at pH 7 and at room temperature. After 5 min of reaction, the mixture was separated by centrifugation (9500 rpm, 10 min). The supernatant was analyzed by UV-vis spectroscopy at a wavelength of 420 nm to monitor the oxidation of pyrogallol. The recovered NMOFtors were washed three times with buffer solution and ethanol before the next catalytic cycle, by adding the same amounts of reactants. The above procedure was repeated for an overall of ten catalytic cycles. The relative activity of the biocatalyst corresponds to the ratio of each cycle’s activity over the activity of the first cycle.

For the storage stability test, 0.2 g/L of the TA-NMOFtors and an equivalent amount of free enzyme were stored at 4 ℃ for different time intervals, and the retained activity of the biocatalyst was assessed following the method described in Section 1.4.

**S1.6 Determination of evolved O_2_**

The O_2_ accumulation in different NMOFtor systems was monitored using a Benchtop Meter with Dissolved Oxygen/RDO Module from Thermo Scientific. Prior to each measurement, the sensor was calibrated using air-saturated deionized water at room temperature (25 ± 1 °C) to ensure accuracy. Gradient experiments were conducted at four H_2_O_2_ concentrations: 0.06%, 0.15%, 0.3%, and 0.6% (v/v). For each test, a specific amount of NMOFtor materials (typically 5 mg) was dispersed in 10 mL of freshly prepared H_2_O_2_ solution in a sealed quartz vessel, maintained at room temperature (25 ± 1°C). Dissolved oxygen levels were recorded at designated time intervals of 0.5, 1, 2, 3, 4, 6, and 10 minutes.

The initial oxygen generation rate was estimated by calculating the slope of the DO versus time curve within the first 60 seconds. All experiments were performed in duplicate, and average values were reported. These DO measurements served as an indirect indicator of catalytic activity and were analyzed in parallel with nanomotor propulsion data to evaluate the relationship between catalytic efficiency and motion behavior under different fuel concentrations.

**S1.7 H_2_O_2_ Decomposition Measurement**

The residual concentration of hydrogen peroxide (H_2_O_2_) was determined using a commercial assay kit (Solebao, BC3595), based on the titanium sulfate colorimetric method.  To initiate the decomposition reaction, 5 mg of enzyme@ZIF-8 nanomotor samples were dispersed in 500 μL of Tris buffer (0.05 M, pH 8.0), and then mixed with 500 μL of hydrogen peroxide solution to achieve a final H_2_O_2_ concentration of 0.15%.

At designated time intervals (30, 60, 90, 120, 180, and 240 s), 50 μL aliquots were withdrawn from each reaction mixture and immediately diluted with pre-chilled acetone to reach a final concentration within the linear detection range (typically ≤1 μmol/mL). Each diluted sample was then mixed sequentially with 25 μL of Reagent II and 50 μL of Reagent III in Eppendorf tubes, vortexed thoroughly, and centrifuged at 4000 g for 10 min at room temperature. After discarding the supernatant, the resulting precipitate was washed three times with acetone, redissolved in 250 μL of Reagent IV. After standing at room temperature for 5 min, 200 μL of each solution was transferred to a 96-well microplate for measurement. The absorbance at 415 nm was recorded using a microplate reader (BMG, POLARstar) and compared to a standard calibration curve (Fig. S13) to calculate H_2_O_2_ concentrations at each time interval.

**S1.8 BPA decontamination and recycling experiments**

The recycling of enzyme@ZIF-8 NMOFtors (virgin and TA engineered) was performed using a reaction mixture containing 0.25 g/L NMOFtors, 5 ppm BPA, and 0.15 wt% H_2_O_2_ in a total volume of 2 ml at pH 7 and room temperature. After a 10-minute reaction period, the mixture was separated by centrifugation (7000 rpm, 5 min). The supernatant was analyzed by LC-MS to quantify the BPA concentration. The recovered NMOFtors were washed three times with water and subsequently used for a second decontamination cycle with equivalent amounts of reactants. The above procedure was repeated for a total of ten cycles. The BPA removal efficiency by different NMOFtors was calculated by measuring the BPA concentration before and after each treatment cycle. The reused NMOFtors were then subjected to PXRD, SEM, and FTIR characterization and product identification (Supporting Information 1.10).

To determine whether enzyme leakage contributed to the observed decrease in BPA removal efficiency during cycling, fluorescence-based leakage assays were performed in parallel with the BPA degradation experiments. FITC-labeled HRP and AF350-labeled CAT (prepared as described in Section 1.3) were encapsulated into ZIF-8 following the same biomineralization procedure. The resulting fluorescent enzyme@ZIF-8 NMOFtors were subjected to identical BPA decontamination cycles as described above.

After each cycle, the supernatant was collected before washing and analyzed by fluorescence spectrophotometry (Ex/Em: FITC 483/520 nm; AF350 346/442 nm). The concentration of released enzymes was quantified using the corresponding calibration curves. The cumulative leakage ratio (%) was calculated by comparing the total released enzyme content in the supernatant solution with the initially encapsulated enzyme amount in the NMOFtors. To further verify the retention of CAT and HRP within the ZIF-8 crystals, the recovered fluorescent enzyme@ZIF-8 NMOFtors after cycling experiments were imaged using a confocal laser scanning microscope.

**S1.****9 Finite element method (FEM) simulations**

The FEM environment of COMSOL Multiphysics (ver. 5.5; COMSOL) was used to test the proposed explanation for the enrichment effect of different species on the surface of enzyme@ZIF-8 NMOFtors and TA-NMOFtors and the diffusion process in MOFtors. Based on the experimental results, enzyme@ZIF-8 NMOFtors and TA-NMOFtors were modeled as polygons with round holes, where the central hole represented the enzyme, and the surrounding holes represented pores with a diameter of 5 nm. The thickness of the ZIF-8 and TA-ZIF-8 shells around the enzyme was set to 60 nm and 200 nm, respectively. In the “Chemical Engineering” module, surface equilibrium reactions on porous MOF shells were defined for the adsorption-desorption of dye species [S6]. The “Transport of Diluted Species” module was used to simulate the concentration diffusion of target species from bulk solution to MOFtor surfaces and the mass transfer in enzyme@ZIF-8 and TA-enzyme@ZIF-8.

**S1.10 Extraction of oxidation products on the NMOFtors**

For product analysis, the separated NMOFtors after reuse cycles was transferred into a glass vial containing 3.0 mL 0.5 M HCl solution, then sealed and incubated for 6 h to dissolve the MOF framework. The solid residue was extracted by a 2-mL mixture of dichloromethane and methanol (1:1, v/v) for 12 h to analyze the possible oxidation products adsorbed on NMOFtors [S7, S8]. This was followed by filtration and then analyzed by UHPLC-MS/MS characterization of the obtained filtrates.

**S1.11 UHPLC-MS/MS analysis**

A Triple quadrupole LC-MS/MS system (QTrap 5500, ABSciex, USA) was utilized for product identification. The chromatographic separation was established by a Waters Acquity HSS-T3 C18 column (2.1 mm × 100 mm × 1.8 μm) with a mobile phase of HPLC-grade acetonitrile and 0.1% formic acid at a ﬂow rate of 0.2 mL·min^-1^ [S9]. The MS/MS system was equipped with an electron spray ionization (ESI) source operating in negative ESI mode (ESI–) for analysis of extracted products. The MS instrumental parameters were set as follows: source temperature, 350 ºC; negative ion spray voltage, -4500 V; entrance potential (EP), -30 V; declustering potential (DP), -40 ∼ -120 V; collision cell exit potential (CXP), -9 ∼ -17 V; collision energy (CE), -20 ∼-100 V. The nebulizer gas (gas 1) and heater gas (gas 2) were set at 50, with curtain gas with a flow rate of 35 arbitrary units. The total ionization chromatography was collected in a mass scan range of *m*/*z* 100‒1200 to identify the conversion products of BPA [S10]. All instruments were controlled and synchronized using Analyst software (version 1.6, AB SCIEX, Foster, CA, USA), which was also employed for data analysis.

Supplementary Figures and Tables


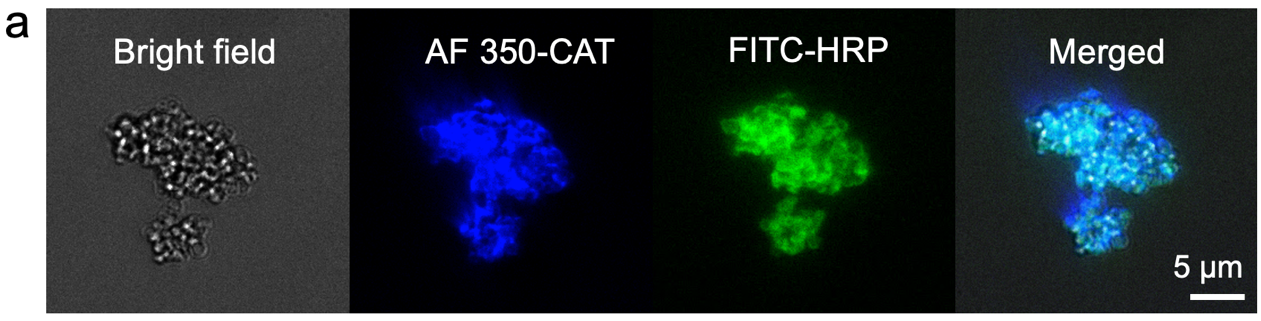


**Fig. S1** CLSM and bright field microscopy images of enzyme@ZIF-8 NMOFtors. CAT and HRP were labelled with AF350 and FITC, respectively (scale bar is 5 μm)


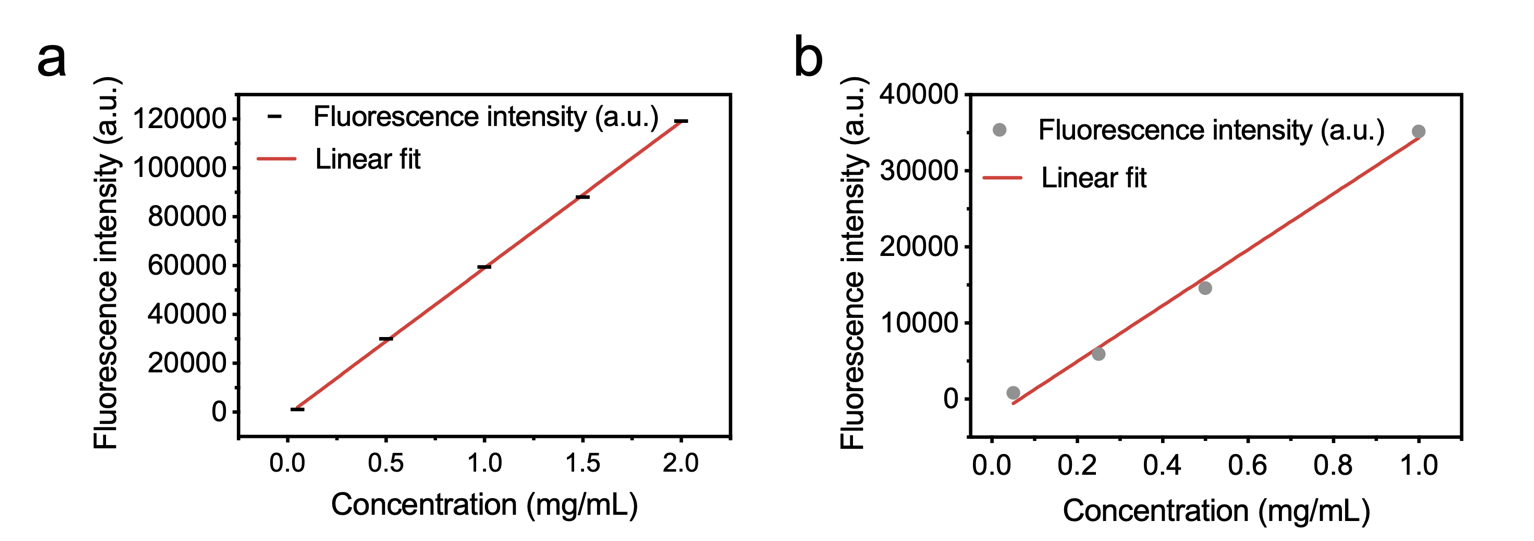


**Fig. S2** **a**) HRP concentration standard curve by measuring FITC-labeled HRP, **b**) CAT concentration standard curve by measuring AF350-labeled CAT using a fluorescence spectrophotometer

**

**Fig. S3** **a**) N_2_ adsorption/desorption isotherms and **b**) pore size distribution of enzyme@ZIF-8 and TA- enzyme@ZIF-8, respectively


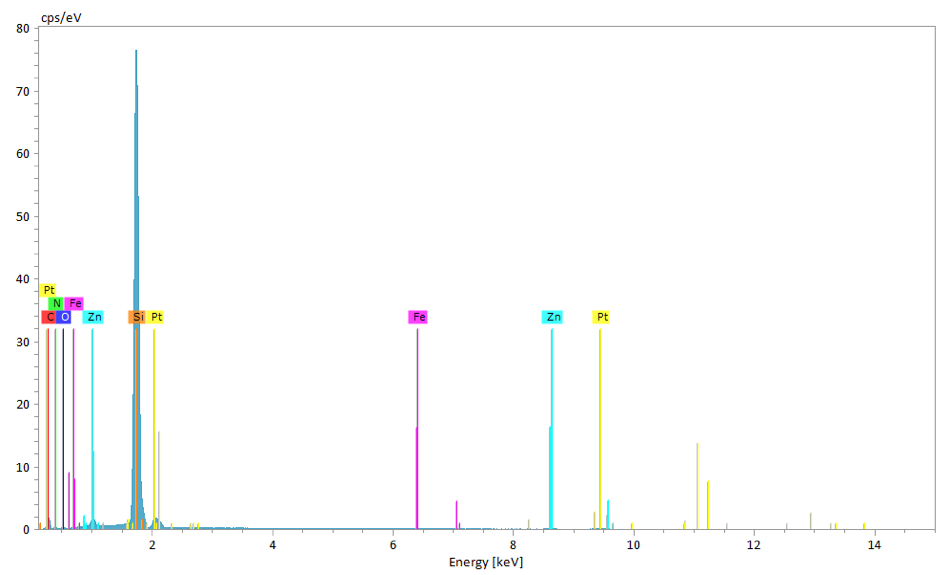


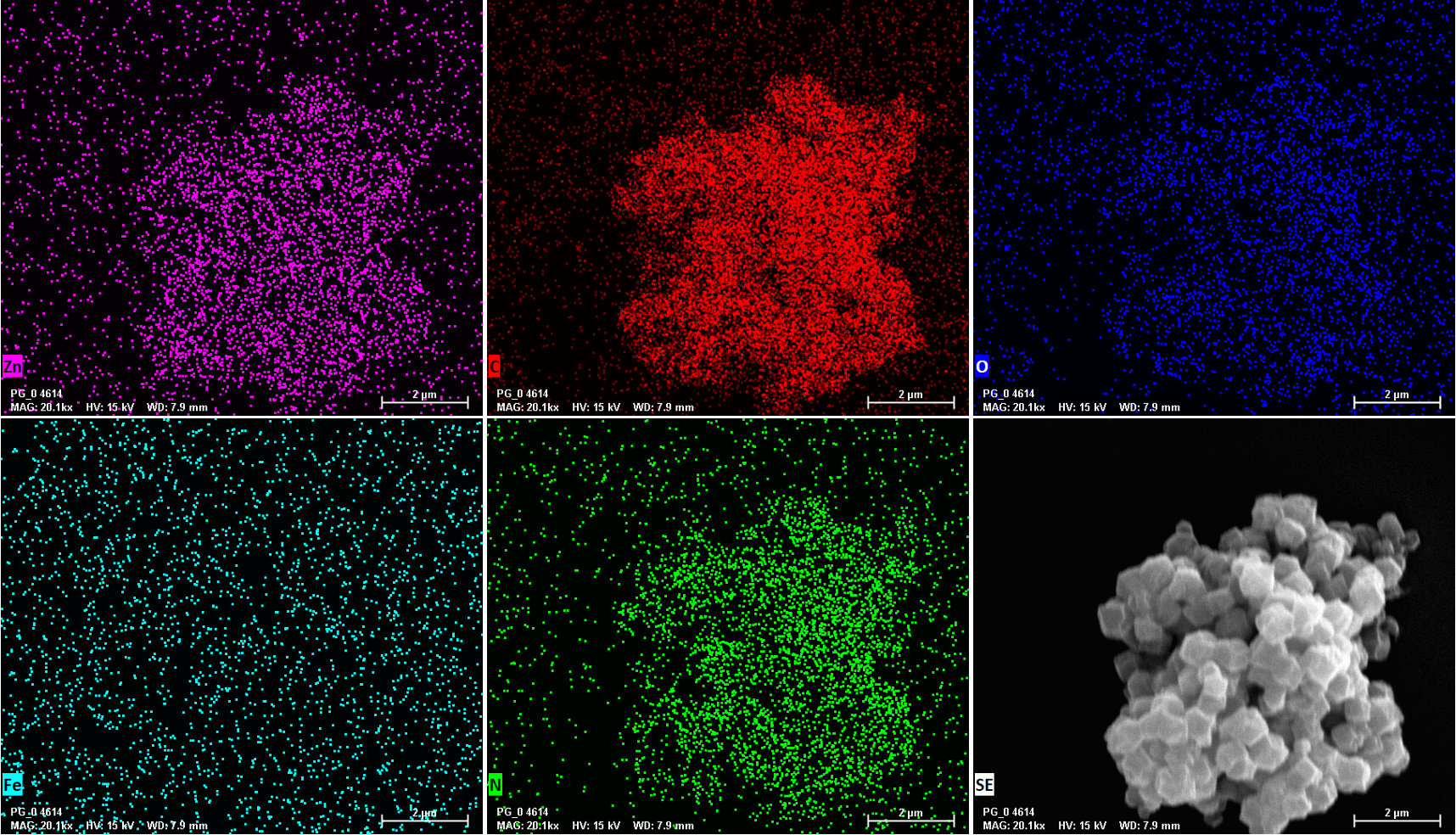


**Fig. S4** SEM/EDS (Elemental Analysis) of enzyme@ZIF-8


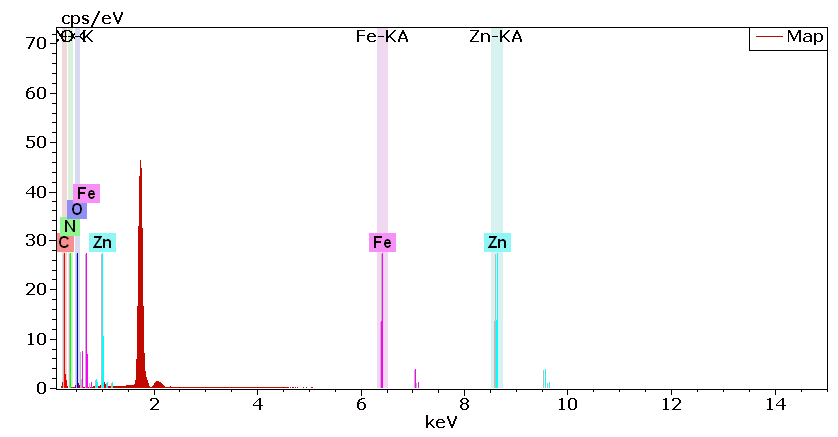


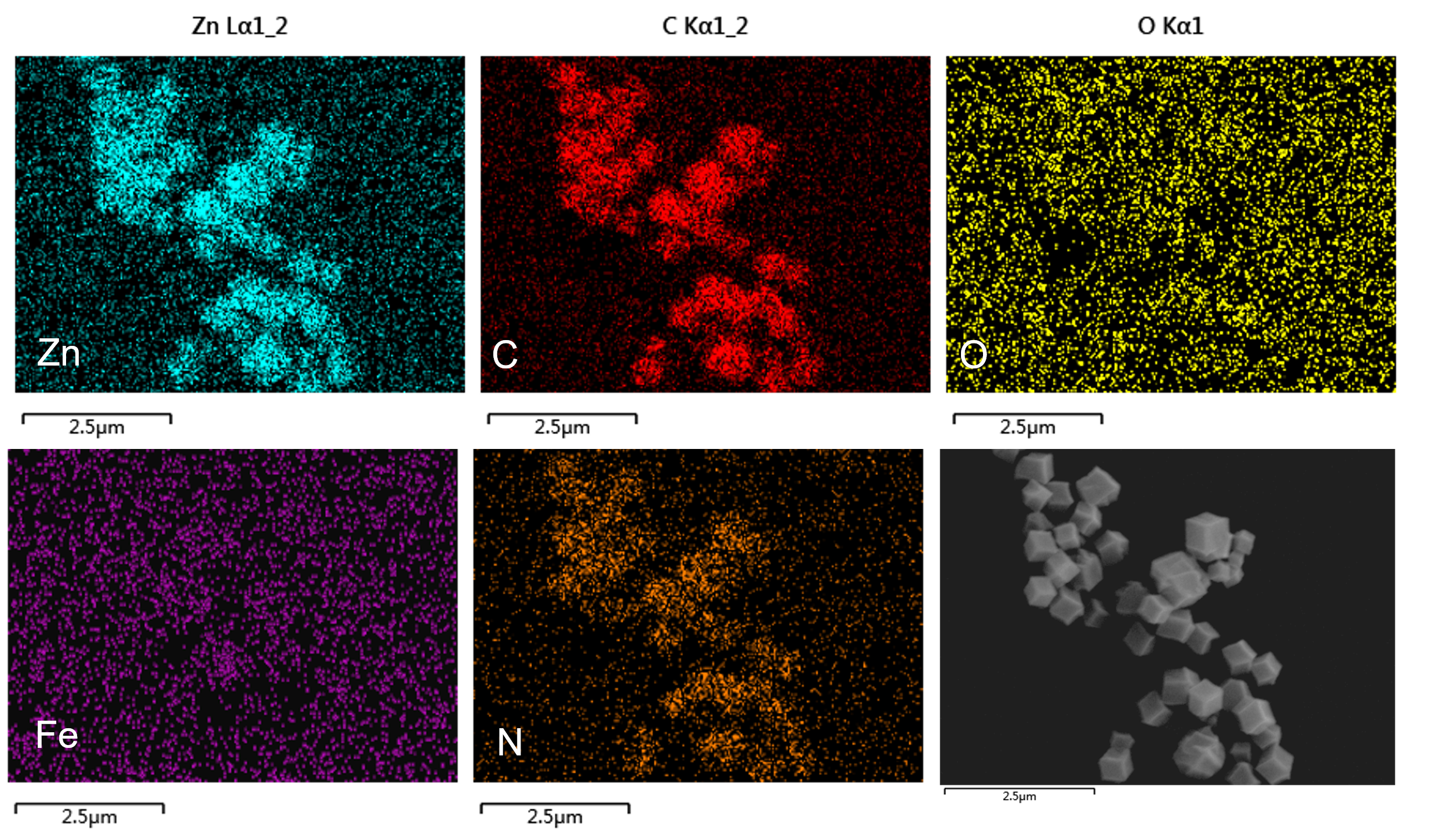


**Fig. S5** SEM/EDS (Elemental Analysis) of TA-treated enzyme@ZIF-8

**
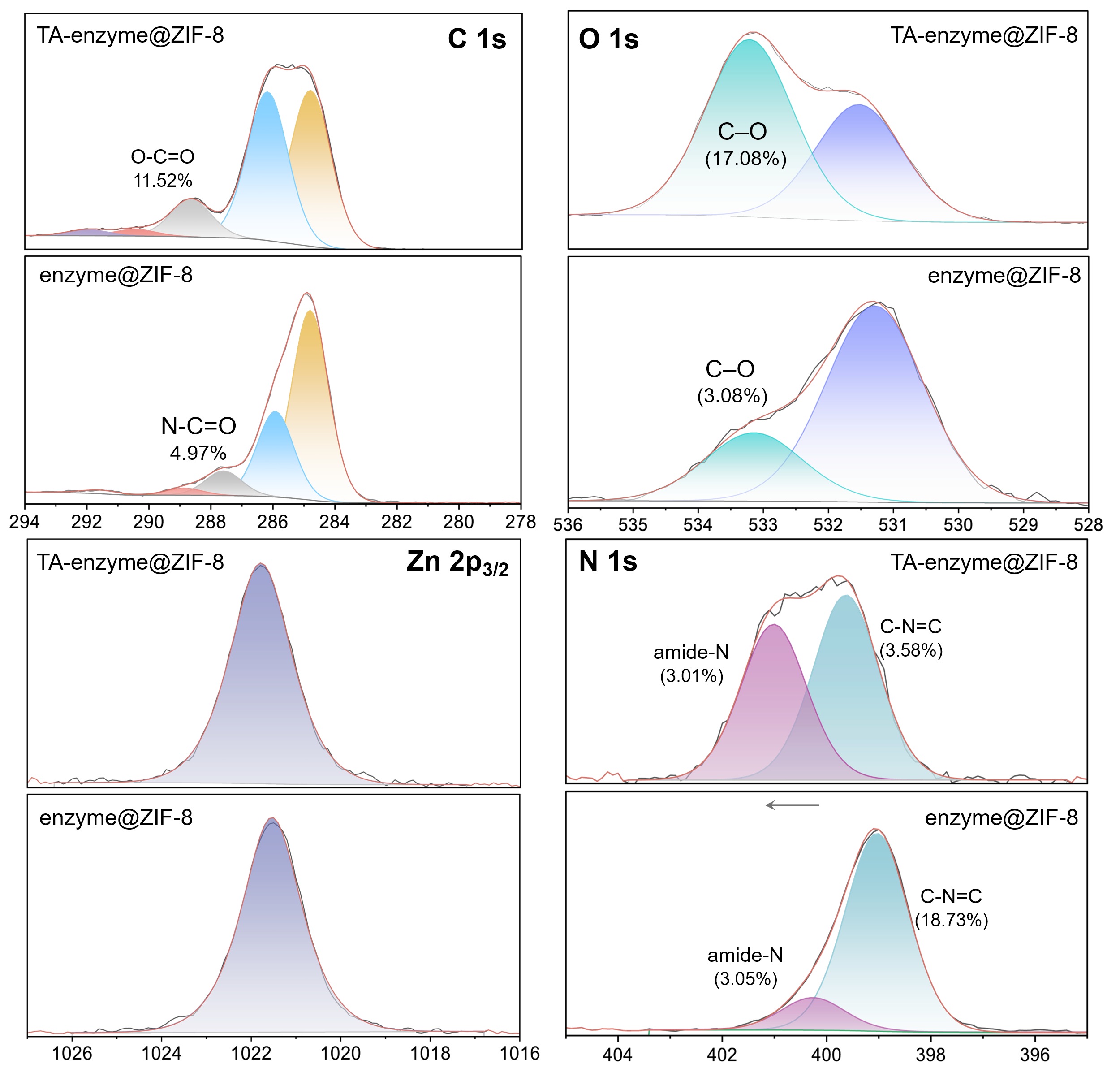
**

**Fig. S6** High-resolution XPS spectra of C 1s, O 1s, N 1s, Zn 2p of enzyme@ZIF-8 and TA- enzyme@ZIF-8


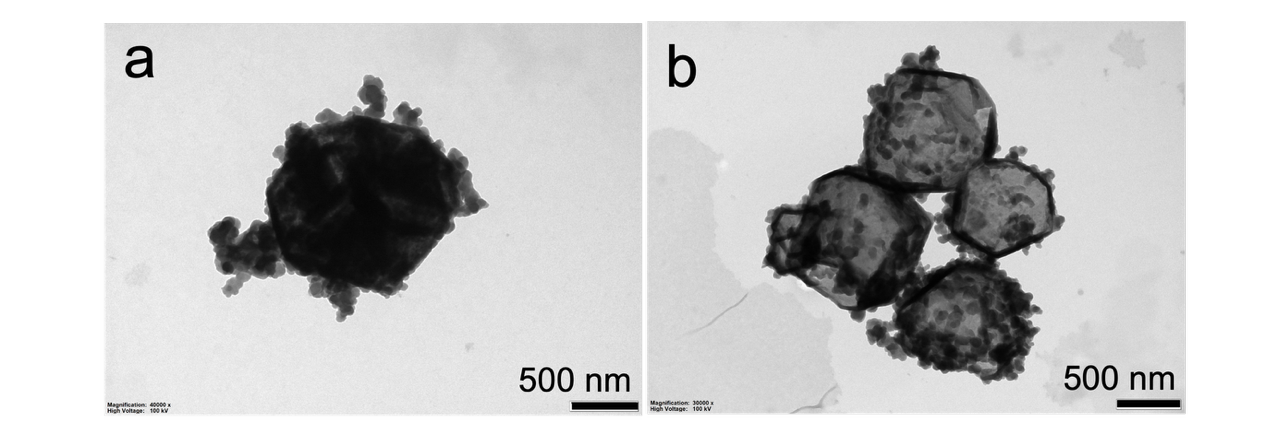


**Fig. S7** TEM images of enzyme@ZIF-8 after etching with TA concentrations of **a**) 2 g/L and **b**) 10 g/L


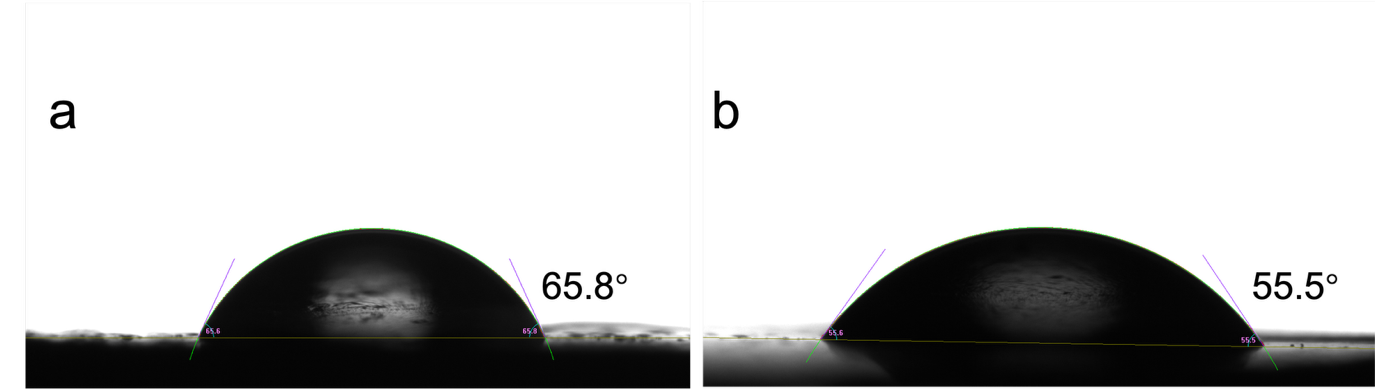


**Fig. S8** Contact angle measurements of enzyme@ZIF-8 before and after TA modification


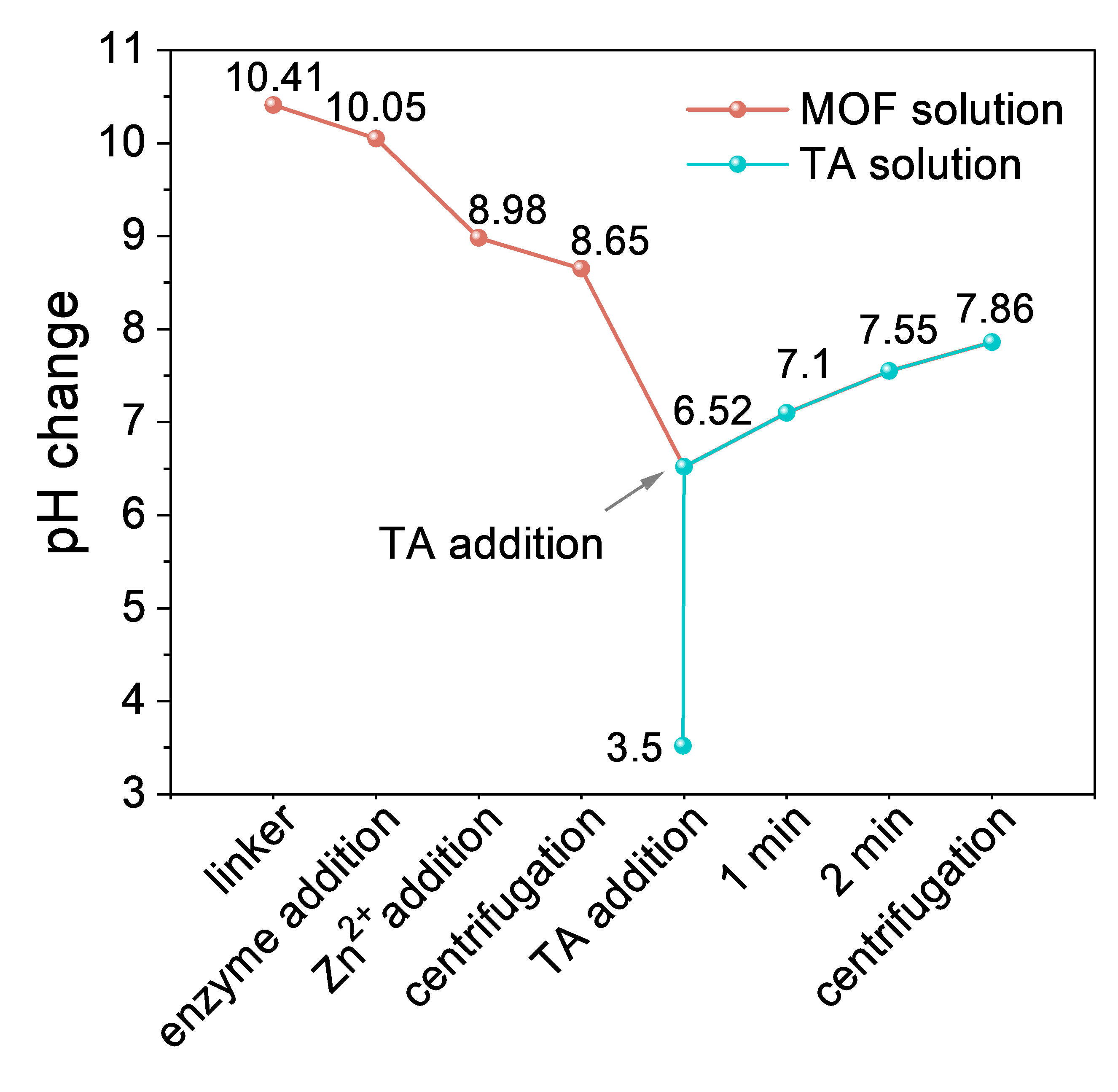


**Fig. S9** The pH changes during the TA-enzyme@ZIF-8 formation

**
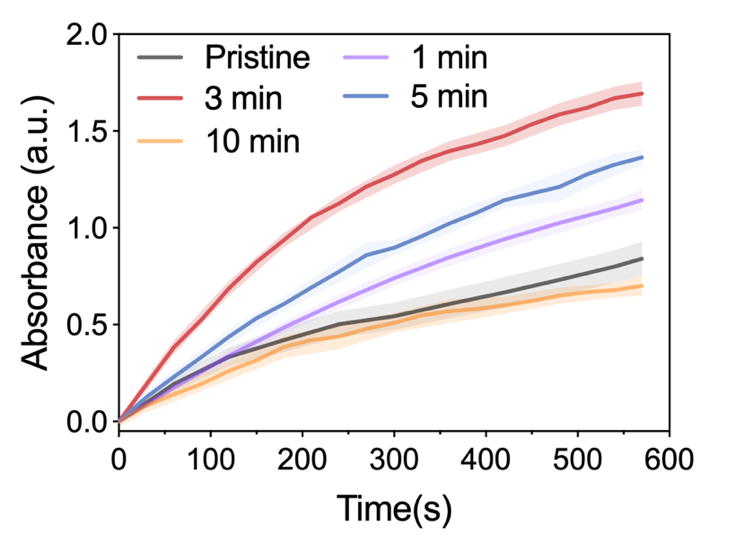
**

**Fig. S10** Time-dependent enzymatic kinetics of TA-NMOFtors treated at different etching times. The TA concentration was 6 g/L


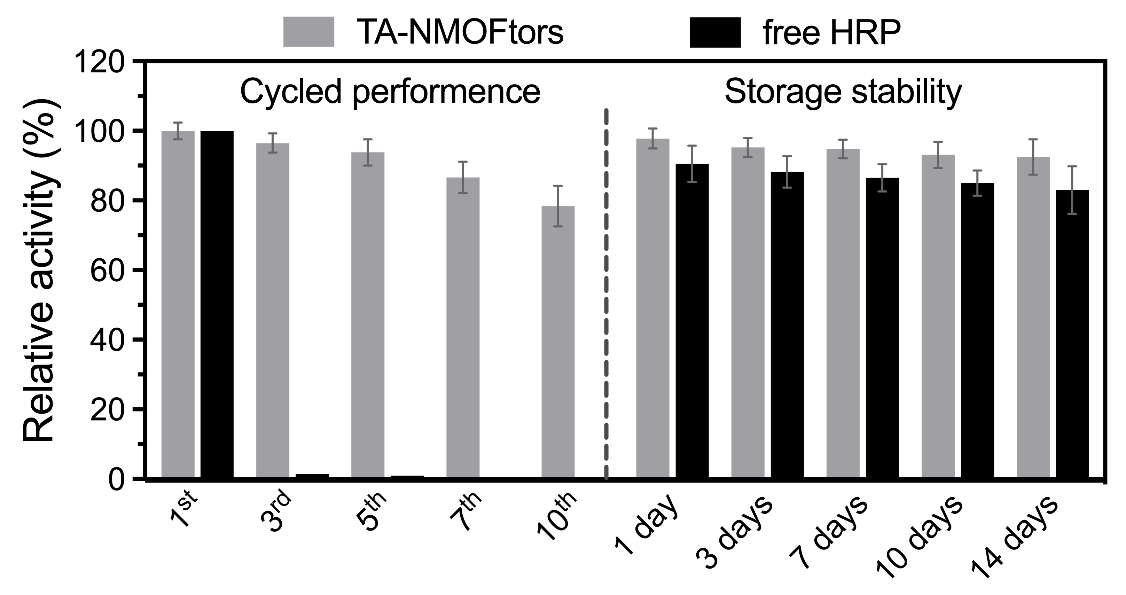


**Fig. S11** Relative catalytic activity of TA-NMOFtors over 10 catalytic cycles, and stability over 2-week storage


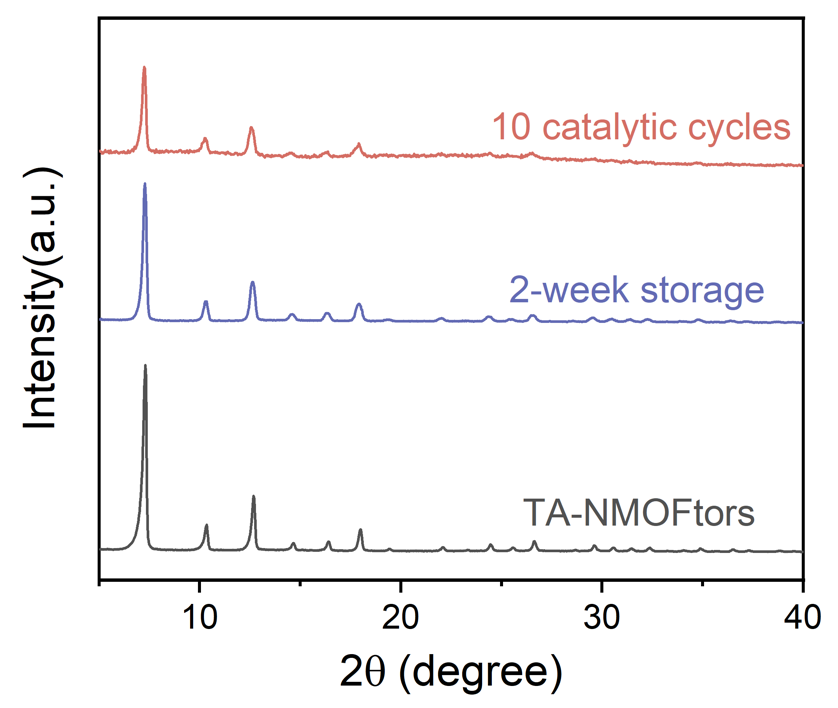


**Fig. S12** PXRD patterns of NMOFtors after catalytic cycles and long-term storage


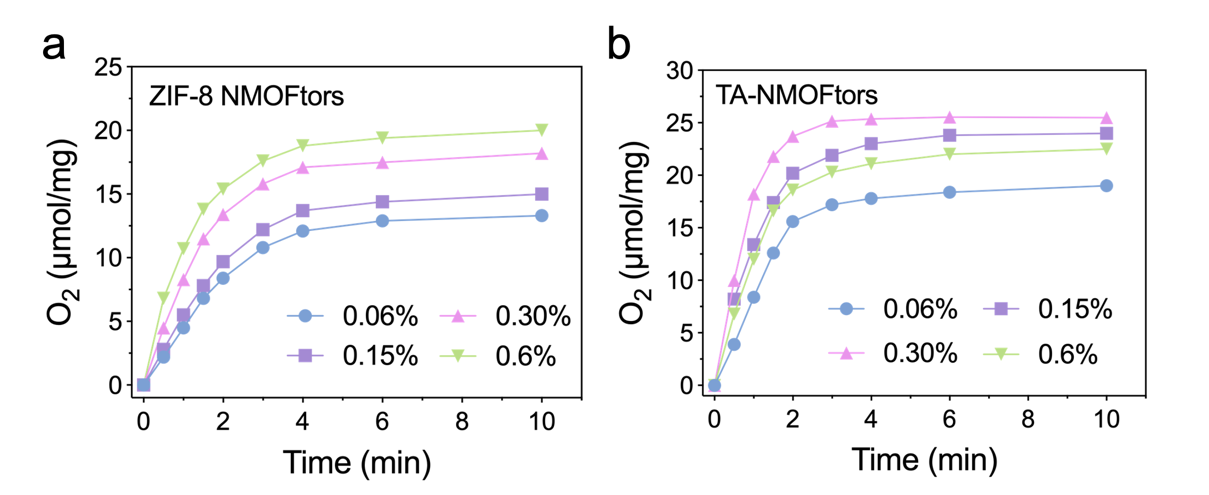


**Fig. S13** Generated O_2_ amount versus time in **a** enzyme@ZIF-8 NMOFtors and **b** TA-NMOFtors with different concentrations of hydrogen peroxide ranging from 0.06% to 0.6%

**
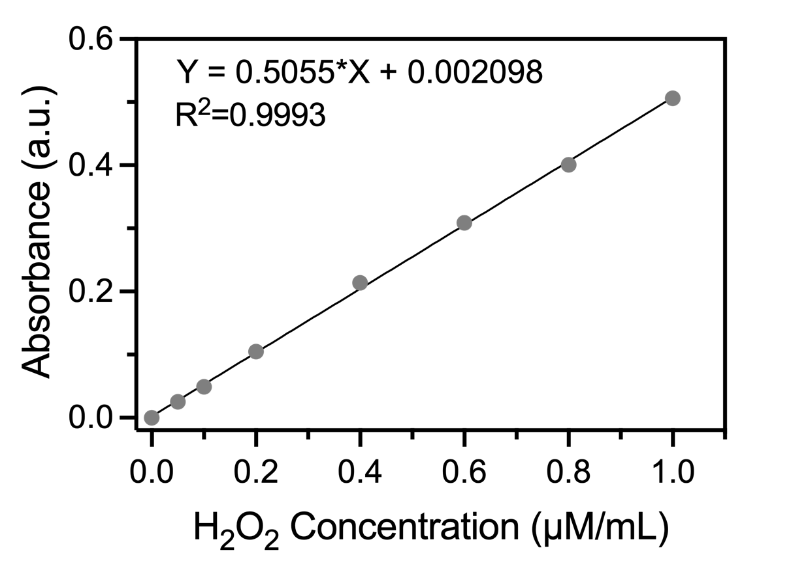
**

**Fig. S14** The calibration curve of the H_2_O_2_ concentration using a titanium sulfate colorimetric assay

**
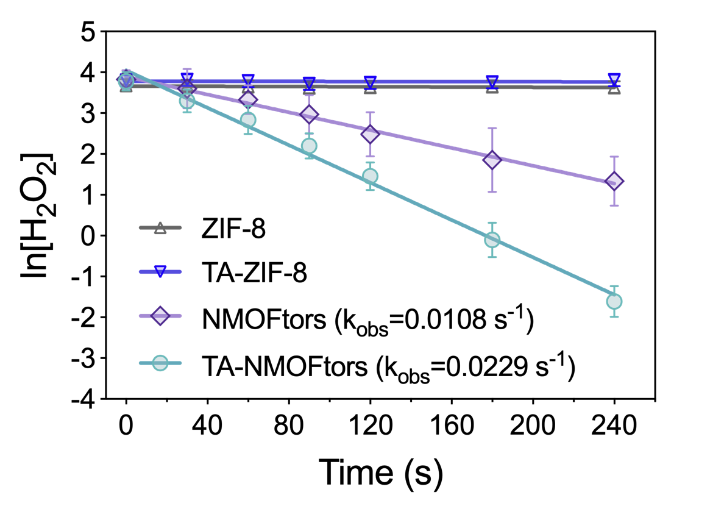
**

**Fig.** **S15** Reaction kinetics of H_2_O_2_ decomposition for ZIF-8, TA-ZIF-8, enzyme@ZIF-8 NMOFtors, and TA-NMOFtors


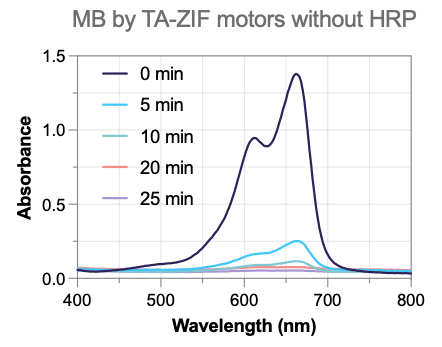


**Fig. S16** Time evolution of the typical UV–vis spectra during MB decontamination by adsorptive TA-CAT@NMOFtors


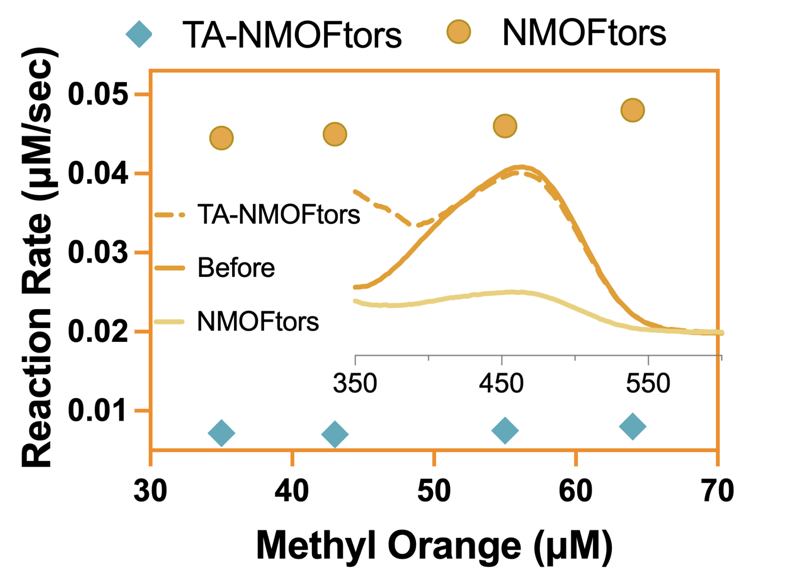


**Fig. S17** Reaction rate of decontamination of MO by enzyme@ZIF-8 NMOFtors (yellow circles) and TA-NMOFtors (blue diamonds). The inset is the corresponding absorbance spectra of MO before and after being treated with NMOFtors


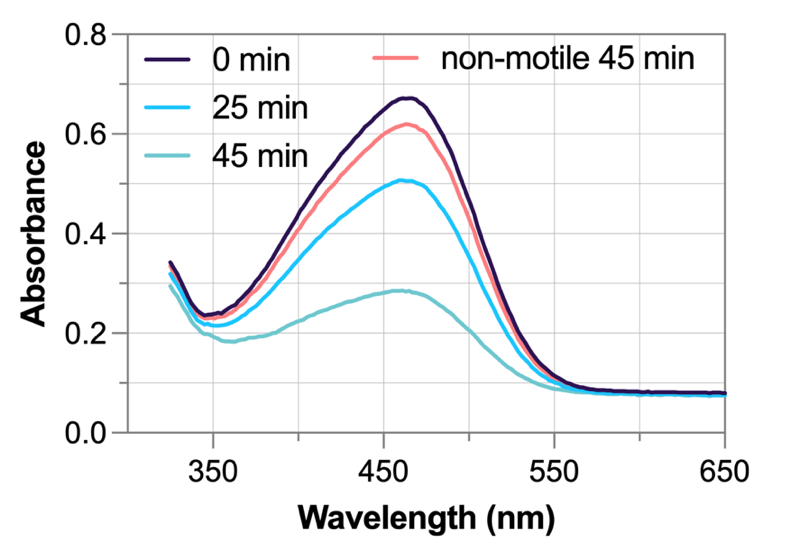


**Fig. S18** The absorbance spectra of MO before and after decontamination by CAT@NMOFtors and non-motile counterparts


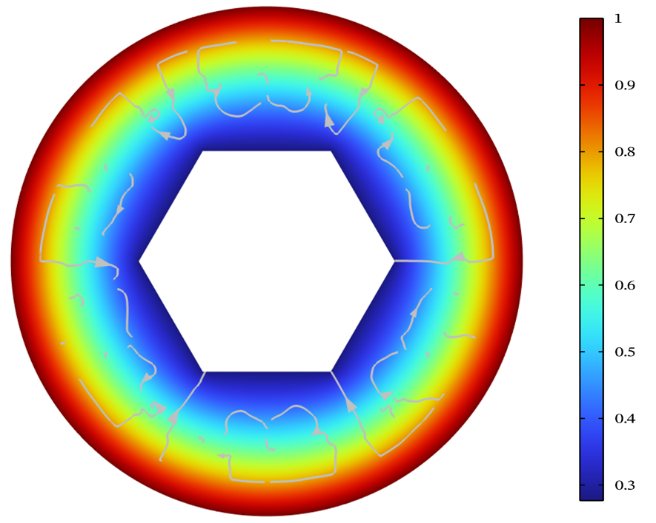


**Fig. S19** Simulated concentration and distribution of local MO on the surface of TA-NMOFtors


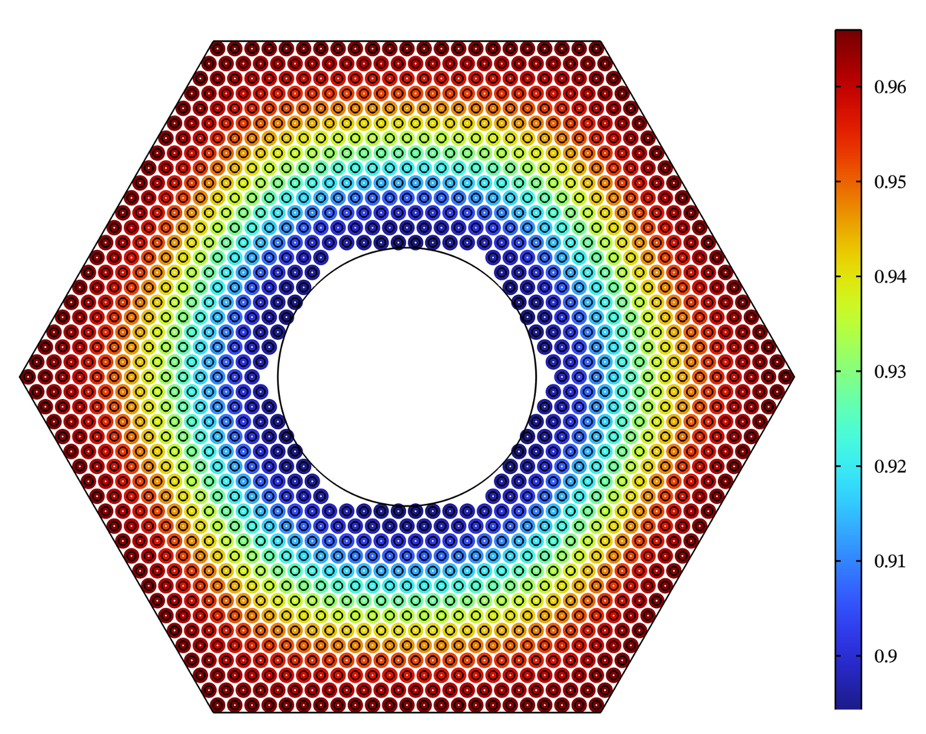


**Fig. S20** Simulation concentration changes on the diffusion pathway due to the adsorption loss at the pore walls in enzyme@ZIF-8 NMOFtors


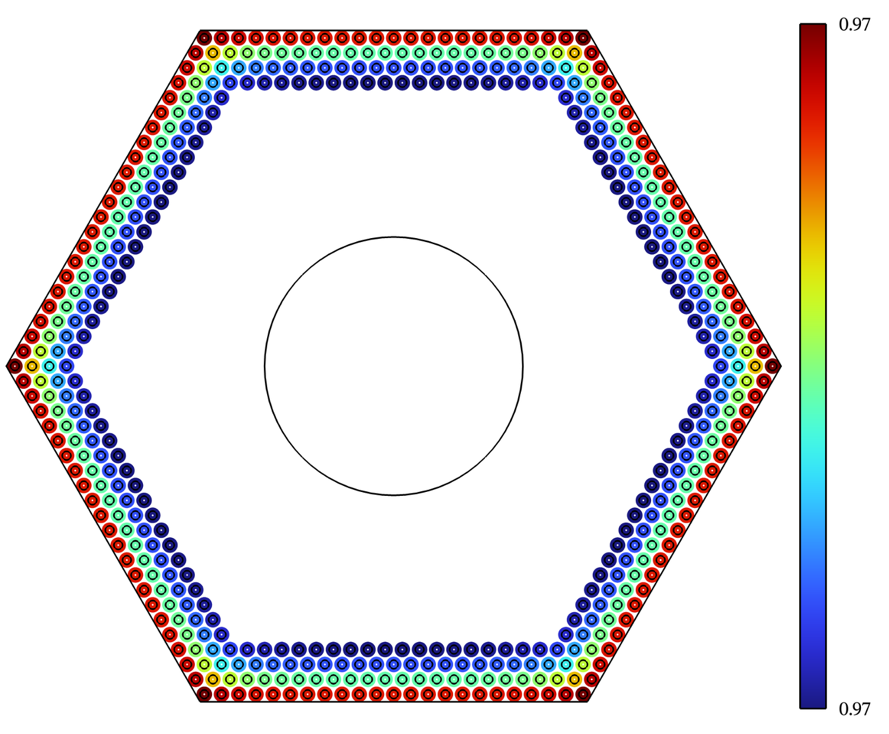


**Fig. S21** Simulation concentration changes along the diffusion pathway due to the adsorption loss at the pore walls in TA-NMOFtors

**Fig. S22 a** Schematic illustration of biocatalytic NMOFtors-based oxidative detoxification of emerging contaminants. **b** BPA removal performance by non-motile (w/o H_2_O_2_), adsorptive, and oxidative enzyme@ZIF-8 NMOFtors and TA-NMOFtors. Error bars represent the standard deviation for three individual recorded tests. **c** Impacts of pH, NOM, and background ions on BPA removal efficiency. **d** Impacts of temperature and real water samples on BPA removal efficiency, and the recycling performance of the NMOFtors for BPA removal

**
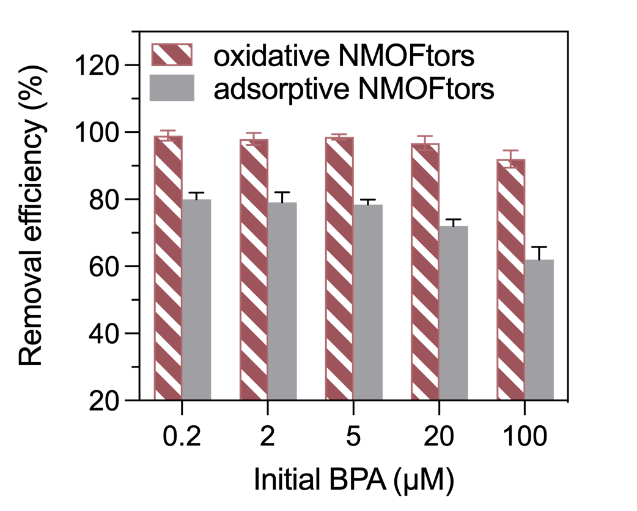
**

**Fig. S23** Removal efficiency of BPA at different initial concentrations in adsorptive and oxidative enzyme@ZIF-8 NMOFtors systems

**
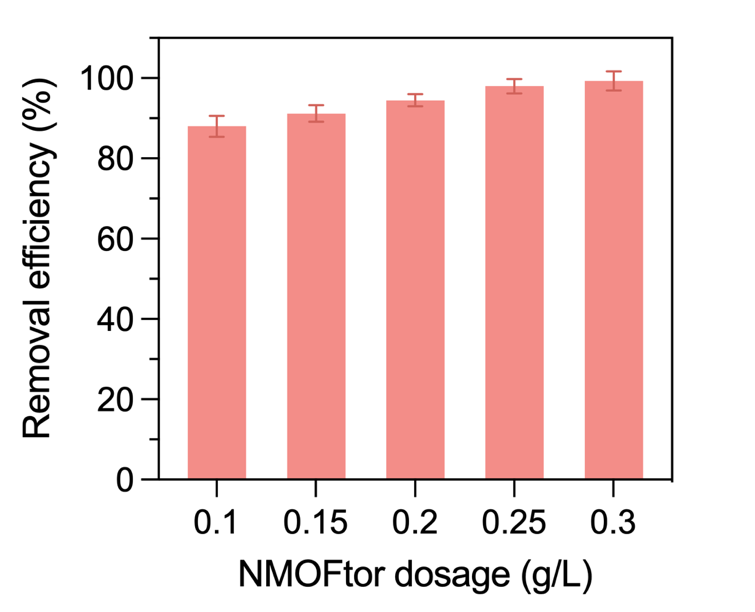
**

**Fig. S24** Effect of TA-NMOFtors dosage on BPA removal efficiency. BPA (20 µM) was treated with varying nanomotor concentrations (0.1, 0.15, 0.2, 0.25, and 0.3 g L^–1^) in the presence of 0.15 % H_2_O_2_ for 2 minutes


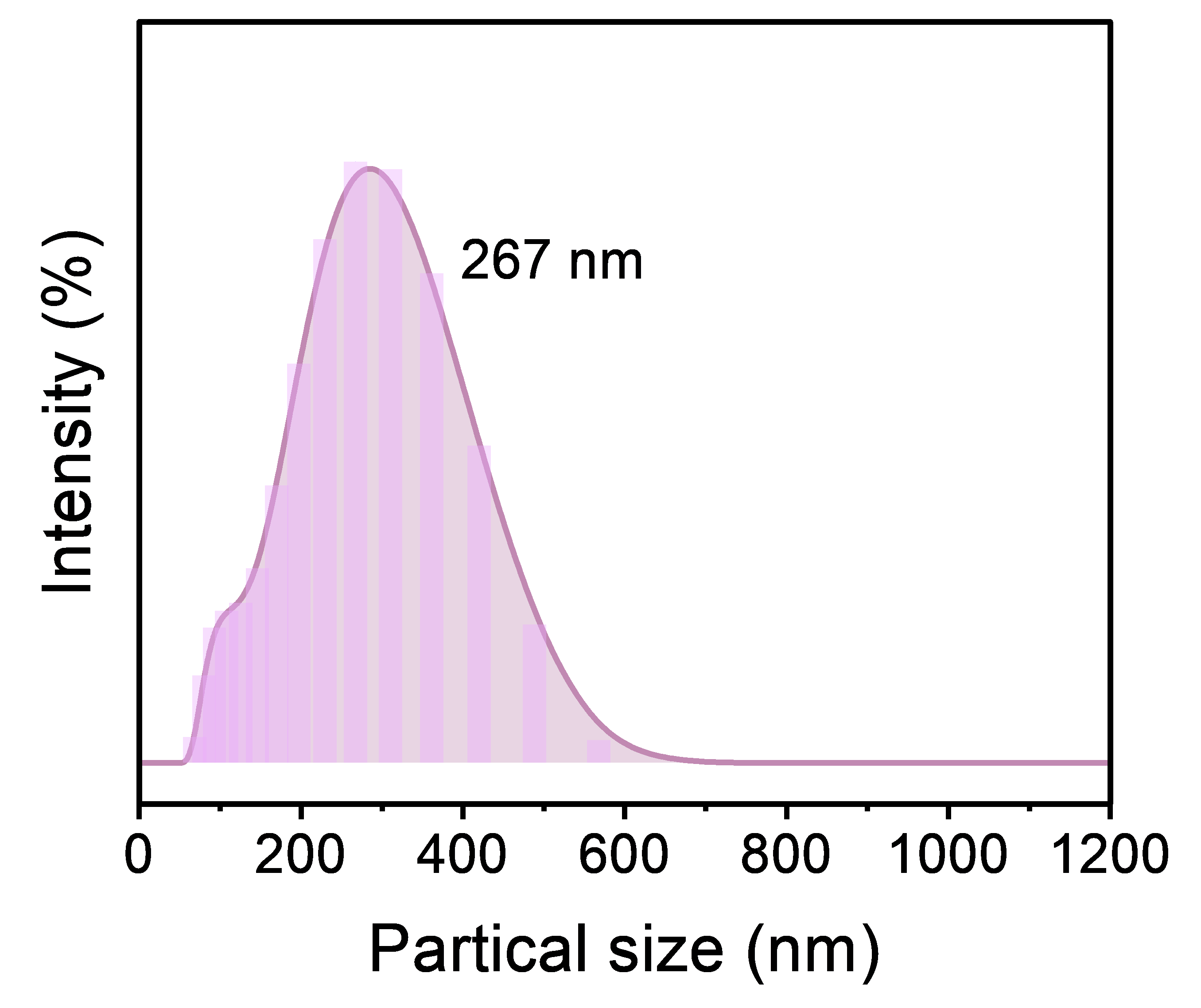


**Fig. S25** The particle size distribution of NOM at pH 7.0

**
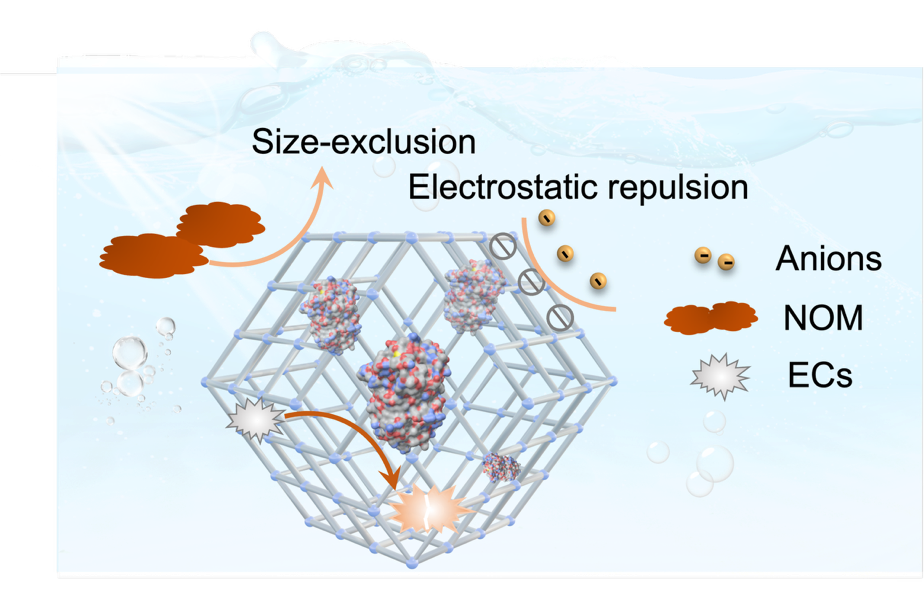
**

**Fig. S26** Schematic mechanism for selective micropollutant removal and resistance to water matrix components in TA-NMOFtors system

**Fig. S27** SEM images and FTIR spectra of enzyme@ZIF-8 NMOFtors after reuse cycles. a) The SEM image shows that the nanomotors largely preserve their rhombic-dodecahedral morphology without observable structural collapse, indicating good mechanical stability during repeated use. b) The FTIR spectra exhibit no significant changes in the characteristic ZIF-8 bands (e.g., 997 cm^–1^) or the enzyme amide I region, confirming that the integrity of both the MOF framework and the enzyme–MOF coordination environment is maintained after reuse

**
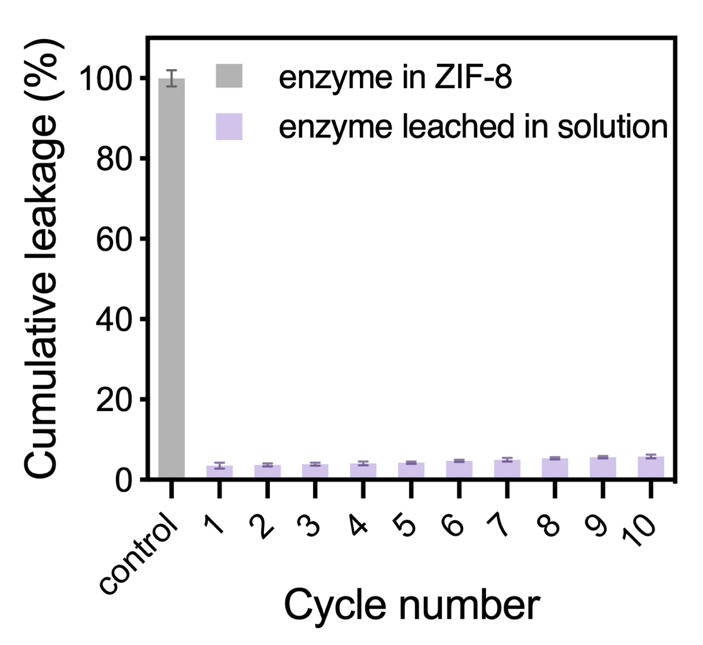
**

**Fig. S28** Fluorescence quantification of labeled enzyme released into the supernatant during catalytic cycles. Cumulative enzyme leakage remained below **6%** of the initially immobilized amount, indicating negligible physical release of CAT/HRP from the ZIF-8 framework

**Fig. S29** Confocal fluorescence microscopy images of fluorescently labeled NMOFtors after recycling experiments. Recovered nanomotors display well-retained and localized fluorescence signal, indicating that the encapsulated enzymes remain confined within the ZIF-8 cavities during operation

**Fig. S30** The HPLC/ESI-MS chromatogram of extracted products after MOFtors treatment after reuse cycles

**Fig. S31** Fragmentation pattern of BPA dimer detected in extracted products after MOFtors treatment after reuse cycles

**Fig. S32** Fragmentation pattern of BPA trimer detected in extracted products after MOFtors treatment after reuse cycles

**Fig. S33** Fragmentation pattern of BPA tetramer detected in extracted products after MOFtors treatment after reuse cycles

**Table S1** Nitrogen isothermal sorption profiles of the pristine and TA-modified samples

| **Sample** | **BET surface area**  **(m² g⁻¹)** | **Total pore volume**  **(cm³ g⁻¹)** |
| --- | --- | --- |
| Enzyme@ZIF-8 | 1116.07 | 0.57 |
| TA-enzyme@ZIF-8 | 783.45 | 0.63 |

**Table S2** Basic characteristics of the different water sources

| **Water sources** | **pH** | **TOC**  **(mgC L-1)** | **Turbidity**  **(NTU)** | **UV254**  **(mg L-1)** |
| --- | --- | --- | --- | --- |
| Tap water | 7.24 | 1.6 | 0.21 | 0.024 |
| Nanjiang River water | 8.17 | 3.6 | 30.5 | 0.095 |

Note: River water and tap water samples were collected from Nanjiang River and Guangzhou Universities Town Campus of South China Normal University, Guangzhou, China.

**Table S3** Comparison of previously reported enzyme-based and MOF-based catalytic systems for water decontamination

| **Category** | **Catalytic system** | **Propulsion Speed**  **(fuel concentration)** | **Micropollutant removal** | **Decontamination**  **mechanism** | **Stability/ Recyclability** | **Ref.** |
| --- | --- | --- | --- | --- | --- | --- |
| Free enzyme | Laccase | None (diffusion-limited) | BPA 48–78% removal in 1–2 h | Enzymatic oxidation | Low stability;  Not recyclable | [S11] |
|  | HRP/H_2_O_2_  (free enzyme) | None (diffusion-limited) | BPA: 90% in 25 min (1.0 unit/mL)  100% in 5 min  (2.5 unit/mL) | Enzymatic oxidation |  | [S8] |
| Immobilized enzyme | MP8@MIL-101(Cr) | None (diffusion-limited) | MO (0.6 μM·s⁻¹)  > MB (0.05 μM·s⁻¹) | Charge-based enrichment | <15% loss after 1 month; 66% after 5 cycles | [S12] |
|  | HRP@ACA-COF | None (diffusion-limited) | MBT, PCM, CA, MPB, FS, SA: 99% in 1 h (with mediator) | Mediator-assisted enzymatic oxidation | High thermal stability  ~60% activity after 5 cycles | [S13] |
|  | MGelMA-CS-Lacs | None (diffusion-limited) | BPA >99% in 6.5 h | Nanopore-assisted biodegradation | 73.7 % by the tenth cycle | [S14] |
|  | Lac@ZIF-8 | None (diffusion-limited) | DFC: 93.5%, NOR: 94.9% in 8 h | Adsorption Biodegradation | 79–83% after 6 cycles | [S15] |
| MOF-based micro/nanomotors | UiO-type catalase micromotor | ~3.56 body length·s⁻¹ (1.5% H_2_O_2_) | RhB 51% | Active adsorption | Not reported | [S16] |
|  | CAT@ZIF-8 nanomotor | 0.27–0.67 mm/s  (0.2 –1%) | Ce²⁺, Cu²⁺, Co²⁺, Mn²⁺, Ni²⁺ (37%–99%); PFOA 91% | Active adsorption | Structurally stable; no recyclability data | [S2] |
|  | MOF-525(Co)–Fe micromotor | ~30 μm·s⁻¹ (0.1%) | Malachite Green  93.55 % in 2 h | Active adsorption  photocatalysis | 73.15 % after 5 cycles | [S17] |
|  | ZnO@ZIF-8/  Fe₃O₄@AgNPs micromotor | 75 to ∼1063 μm·s⁻¹  (5% –30% H_2_O_2_  and 1.5% SDS) | RhB dye  98.6% in 60 min | Active adsorption  photocatalysis | 92.6% for 2 cycles | [S18] |
|  | CAT/HRP@ZIF-8 NMOFtors | 0.796 mm/s (0.15% H_2_O_2_)  1.13 mm/s (0.3% H_2_O_2_) | MB: 98.7% in 5 min; BPA: 98% in 2 min | Active adsorption  Charge-based enrichment  Enzymatic transformation | <10% loss after 2 weeks; >80% after 10 cycles | Tthis  wwork |

* Abbreviations: MBT (2-mercaptobenzothiazole); PCM (paracetamol); CA (caffeic acid); MPB (methyl paraben); FS (furosemide); SMO (sulfamethoxazole); SA (salicylic acid); DFC (diclofenac) and NOR (norfloxacin).

Supplementary References

1. K. Liang, R. Ricco, C.M. Doherty, M.J. Styles, S. Bell et al., Biomimetic mineralization of metal-organic frameworks as protective coatings for biomacromolecules. Nat. Commun. **6**, 7240 (2015). <https://doi.org/10.1038/ncomms8240>
2. Z. Guo, J. Liu, Y. Li, J.A. McDonald, M.Y. Bin Zulkifli et al., Biocatalytic metal-organic framework nanomotors for active water decontamination. Chem. Commun. **56**(94), 14837–14840 (2020). <https://doi.org/10.1039/d0cc06429g>
3. J. Liu, J. Xue, L. Fu, J. Xu, M.S. Lord et al., Genetically encoded synthetic beta cells for insulin biosynthesis and release under hyperglycemic conditions. Adv. Funct. Mater. **32**(18), 2111271 (2022). <https://doi.org/10.1002/adfm.202111271>
4. F.-K. Shieh, S.-C. Wang, C.-I. Yen, C.-C. Wu, S. Dutta et al., Imparting functionality to biocatalysts *via* embedding enzymes into nanoporous materials by a *de novo* approach: size-selective sheltering of catalase in metal–organic framework microcrystals. J. Am. Chem. Soc. **137**(13), 4276–4279 (2015). <https://doi.org/10.1021/ja513058h>
5. W. Liang, H. Xu, F. Carraro, N.K. Maddigan, Q. Li et al., Enhanced activity of enzymes encapsulated in hydrophilic metal–organic frameworks. J. Am. Chem. Soc. **141**(6), 2348–2355 (2019). <https://doi.org/10.1021/jacs.8b10302>
6. A. Li, X. Qiao, K. Liu, W. Bai, T. Wang, Hollow metal organic framework improves the sensitivity and anti-interference of the detection of exhaled volatile organic compounds. Adv. Funct. Mater. **32**(30), 2202805 (2022). <https://doi.org/10.1002/adfm.202202805>
7. X. Gao, Z. Yang, W. Zhang, B. Pan, Carbon redirection *via* tunable Fenton-like reactions under nanoconfinement toward sustainable water treatment. Nat. Commun. **15**, 2808 (2024). <https://doi.org/10.1038/s41467-024-47269-6>
8. Q. Huang, W.J. Weber, Transformation and removal of bisphenol A from aqueous phase *via* peroxidase-mediated oxidative coupling reactions:   efficacy, products, and pathways. Environ. Sci. Technol. **39**(16), 6029–6036 (2005). <https://doi.org/10.1021/es050036x>
9. L. Cui, Y. Gong, S. Zhao, Y. Wu, A. Wang et al., Homogenous oxidizing oligomerization coupled with coagulation for water purification. Water Res. **257**, 121684 (2024). <https://doi.org/10.1016/j.watres.2024.121684>
10. K. Sun, Q. Liu, J. Liu, S. Li, X. Qi et al., New insights into humic acid-boosted conversion of bisphenol A by laccase-activated co-polyreaction: Kinetics, products, and phytotoxicity. J. Hazard. Mater. **436**, 129269 (2022). <https://doi.org/10.1016/j.jhazmat.2022.129269>
11. D. Daâssi, A. Prieto, H. Zouari-Mechichi, M.J. Martínez, M. Nasri et al., Degradation of bisphenol A by different fungal laccases and identification of its degradation products. Int. Biodeterior. Biodegrad. **110**, 181–188 (2016). <https://doi.org/10.1016/j.ibiod.2016.03.017>
12. E. Gkaniatsou, C. Sicard, R. Ricoux, L. Benahmed, F. Bourdreux et al., Enzyme encapsulation in mesoporous metal–organic frameworks for selective biodegradation of harmful dye molecules. Angew. Chem. Int. Ed. **57**(49), 16141–16146 (2018). <https://doi.org/10.1002/anie.201811327>
13. N. Elmerhi, K. Al-Maqdi, K. Athamneh, A.K. Mohammed, T. Skorjanc et al., Enzyme-immobilized hierarchically porous covalent organic framework biocomposite for catalytic degradation of broad-range emerging pollutants in water. J. Hazard. Mater. **459**, 132261 (2023). <https://doi.org/10.1016/j.jhazmat.2023.132261>
14. M. Du, J. Liu, B. Huang, Q. Wang, F. Wang et al., Spatial nanopores promote laccase degradation of bisphenol A and its analogs. Sci. Total Environ. **901**, 166429 (2023). <https://doi.org/10.1016/j.scitotenv.2023.166429>
15. M. Lungelo Dlamini, M. Lesaoana, I.A. Kotzé, H.L. Richards, Response surface methodology mediated optimization of diclofenac and norfloxacin biodegradation using laccase immobilized on metal–organic frameworks. Sep. Purif. Technol. **326**, 124709 (2023). <https://doi.org/10.1016/j.seppur.2023.124709>
16. Y. Yang, X. Arqué, T. Patiño, V. Guillerm, P.-R. Blersch et al., Enzyme-powered porous micromotors built from a hierarchical micro- and mesoporous UiO-type metal–organic framework. J. Am. Chem. Soc. **142**(50), 20962–20967 (2020). <https://doi.org/10.1021/jacs.0c11061>
17. D. Lan, J. Xue, Q. Chen, P. Deng, H. Zong et al., Self-propelled MOF nanomotors for dual-function capture and photocatalytic removal of organic contaminant in water. Sustain. Mater. Technol. **43**, e01324 (2025). <https://doi.org/10.1016/j.susmat.2025.e01324>
18. L. Chen, M.-J. Zhang, S.-Y. Zhang, L. Shi, Y.-M. Yang et al., Simple and continuous fabrication of self-propelled micromotors with photocatalytic metal–organic frameworks for enhanced synergistic environmental remediation. ACS Appl. Mater. Interfaces **12**(31), 35120–35131 (2020). <https://doi.org/10.1021/acsami.0c11283>
